# Capillary regression leads to sustained local hypoperfusion by inducing constriction of upstream transitional vessels

**DOI:** 10.1073/pnas.2321021121

**Published:** 2024-09-05

**Authors:** Stephanie K. Bonney, Cara D. Nielson, Maria J. Sosa, Orla Bonnar, Andy Y. Shih

**Affiliations:** ^a^Center for Developmental Biology and Regenerative Medicine, Seattle Children’s Research Institute, Seattle, WA 98101; ^b^Graduate Program in Neuroscience, University of Washington, Seattle, WA 98195; ^c^MassGeneral Institute for Neurodegenerative Disease, Massachusetts General Hospital and Harvard Medical School, Boston, MA 02129; ^d^Department of Pediatrics, University of Washington, Seattle, WA 98195; ^e^Department of Bioengineering, University of Washington, Seattle, WA 98195

**Keywords:** capillary, cerebral blood flow, pericyte, microbleed, two-photon imaging

## Abstract

In the brain, a microvascular sensory web coordinates oxygen delivery to regions of neuronal activity. This involves a dense network of capillaries that send conductive signals upstream to feeding arterioles to promote vasodilation and blood flow. Although this process is critical to the metabolic supply of healthy brain tissue, it may also be a point of vulnerability in disease. Deterioration of capillary networks is a feature of many neurological disorders and injuries and how this web is engaged during vascular damage remains unknown. We performed in vivo two-photon microscopy on young adult mural cell reporter mice and induced focal capillary injuries using precise two-photon laser irradiation of single capillaries. We found that ~59% of the injuries resulted in regression of the capillary segment 7 to 14 d following injury, and the remaining repaired to reestablish blood flow within 7 d. Injuries that resulted in capillary regression induced sustained vasoconstriction in the upstream arteriole-capillary transition (ACT) zone at least 21 days postinjury in both awake and anesthetized mice. The degree of vasomotor dynamics was chronically attenuated in the ACT zone consequently reducing blood flow in the ACT zone and in secondary, uninjured downstream capillaries. These findings demonstrate how focal capillary injury and regression can impair the microvascular sensory web and contribute to cerebral hypoperfusion.

## Significance

Deterioration of the capillary network is a characteristic of many neurological diseases and can exacerbate neuronal dysfunction and degeneration due to poor blood perfusion. Here, we show that focal capillary injuries can induce vessel regression and elicit sustained vasoconstriction in upstream transitional vessels that branch from cortical penetrating arterioles. This reduces blood flow to broader, uninjured regions of the same microvascular network. These findings suggest that widespread and cumulative damage to brain capillaries in neurological disease may contribute more broadly to cerebral hypoperfusion through their remote actions.

Vascular contributions to cognitive impairment and dementia (VCID) include small, but wide-spread ischemic and hemorrhagic injuries called microinfarctions and microbleeds, respectively (1, 2). These lesions are <3 mm in size and may only occupy 1 to 2 mL of the total brain volume. However, their remote effects can depress neural function and contribute more broadly to brain dysfunction and cognitive decline (3). While these microlesions are well-known features of VCID and believed to result largely from pathologies of brain arterioles, even smaller disruptions occur in capillaries, the finest and most delicate vessels in the brain. Capillaries are the site of amyloid beta buildup during Type 1 cerebral amyloid angiopathy, a form of small vessel disease (4), and reactive oxygen species induced by amyloid beta toxicity can cause damage to the capillary endothelium and neighboring pericytes (5). Microbleeds can occur in the absence of capillary amyloid and may result from proteolytic degradation of the endothelium by pericytes or trapped neutrophils (6–8). These events contribute to capillary network rarefaction, as commonly seen in aging and VCID (9, 10), and evade detection by clinical imaging due to their exceedingly small size. Yet, they likely contribute to disease progression in covert and insidious ways. Since the capillary network is dense and redundant in its connectivity (11, 12), it is unclear whether focal capillary changes could lead to broader, yet unrecognized impairments of brain perfusion.

Capillaries sense neuronal activity via Kir2.1 and TRPA1/Panx1 channels in the endothelium (13, 14) and possibly through Kir2.2 on pericytes (15). Both the activity of neurons and astrocytes could lead to release of K^+^ in the vicinity of cerebral microvessels (16). This initiates a capillary-to-arteriole conductive hyperpolarization that is propagated to upstream arteriole-capillary transition (ACT) vessels and penetrating arterioles (PAs) leading to reduced intracellular Ca^2+^, mural cell relaxation, and increased blood flow back downstream into the capillary networks (17). In the retina, the conductive properties of the microvasculature have also been shown to be dependent upon endothelial and pericyte gap junctions (18). The ACT zone is where many vasomodulating signals converge, making it an important locus for blood flow control (19–21). Vessel segments in this region are surrounded by α-smooth muscle actin-expressing ensheathing pericytes and may also include precapillary sphincters at upstream branch points. The ACT zone is highly sensitive to brain activity and is the first to dilate during neurovascular coupling (22, 23). However, it is also susceptible to pathology and exhibits sustained constriction after transient cerebral ischemia (24). Critically, depolarizing electrical stimulation of capillary pericytes leads to a conductive wave of Ca^2+^ increase upstream that elicits robust vasoconstriction in ACT vessels (18). These findings cast the capillary network as a “sensory web” that responds to physiological activity, but also potentially to pathophysiological stimuli.

To test this possibility, we used in vivo two-photon imaging in the mouse cerebral cortex to induce precise capillary injuries, and then tracked the consequence of these injuries over weeks in both anesthetized and awake mice. We asked: 1) Does capillary injury affect dynamics of upstream ACT and PA zones? 2) Are there lasting effects of capillary injury on local blood flow?

## Results

### Focal Capillary Injury Induces Vessel Regression.

**Fig. 1.**
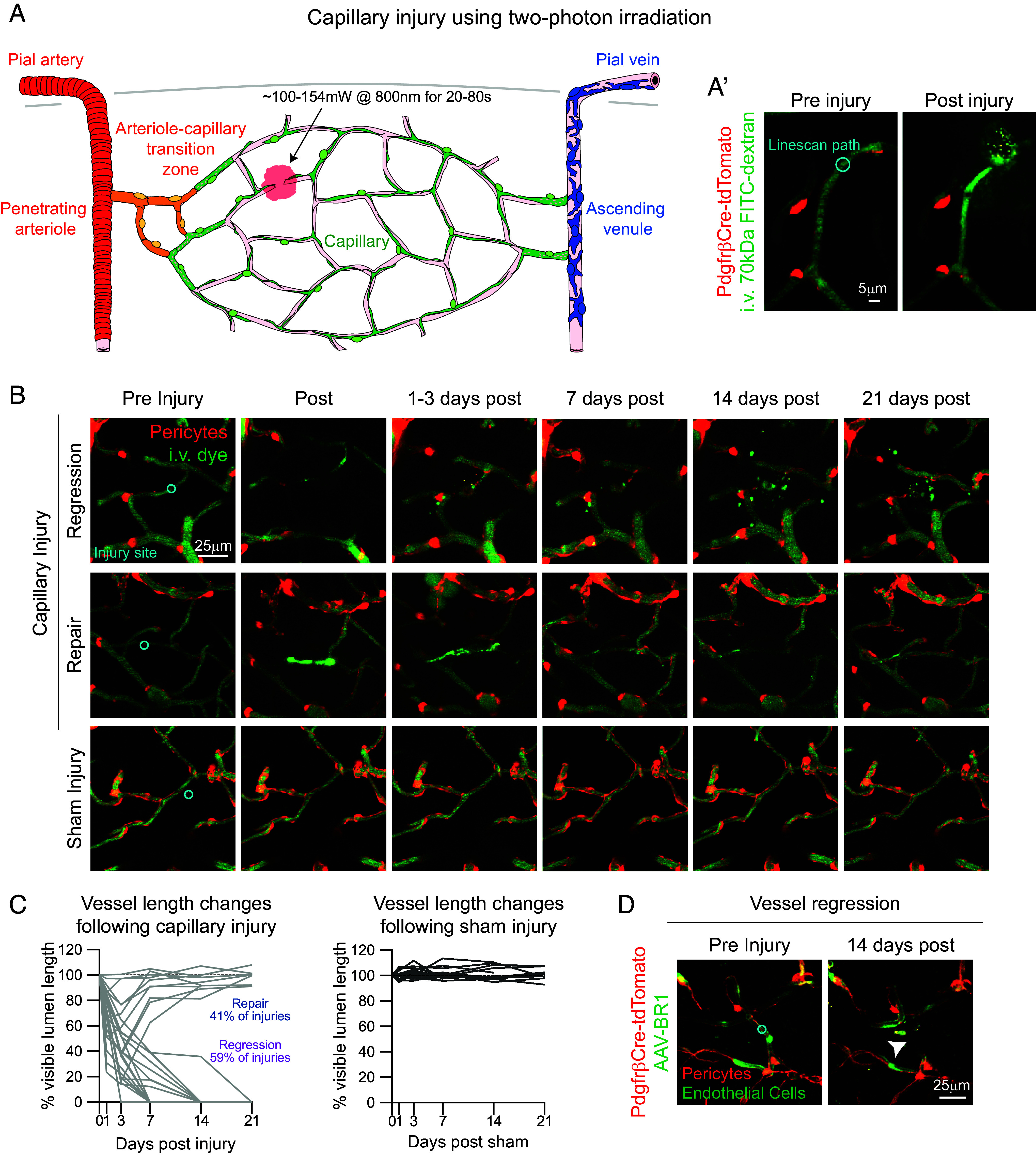
Focal capillary injury induced by optical laser ablation. (*A*) Schematic of a cortical microvascular network with location of two-photon laser-induced capillary injury. Microvascular zones are highlighted including the pial artery and PA (red), arteriole-capillary transition zone (orange), capillary zone (green), AV and pial vein (blue). (*A’*) Representative in vivo image of a capillary injury in a PdgfrβCre-tdTomato mouse preinjury with line-scan path (cyan) and ~10 min postinjury. Pericytes are shown in red and i.v. dye (70 kDa FITC-Dextran) labeling vessels in green. (*B*) Representative in vivo images of a capillary regression, repair, and sham event pre- and postinjury (~10 min), and then at various days postinjury. (*C*) Graphs of percent change in length of the injured (gray; *Left*) or sham (black; *Right*) capillaries based on visible i.v. dye in the lumen preinjury (0 d) and various days postinjury. Overall, 12/29 injuries (41%) resulted in capillary repairs and 17/29 (59%) resulting in regressions, in experiments conducted over 15 mice. Sham injuries = 17 conducted over 13 mice. (*D*) Representative in vivo images of endothelial cells after capillary regression. In 3/3 regression experiments, GFP+ endothelial labeling was no longer present 14 dpi. Pericytes are shown in red and endothelial cells are shown in green.

To understand how capillary injuries affect the microvascular network, we performed in vivo two-photon imaging on mural cell reporter mice (PdgfrβCre-tdTomato) (25) and used a modified model of focal capillary injury involving rupture of the vascular wall (26). Cortical capillaries were identified by distinguishing thin-strand and mesh pericytes on capillary vessels from morphologically distinguished ensheathing pericytes on ACT vessels (19). In general, ruptured capillary segments were 5 to 8 branch orders from PAs. Injuries were induced by focusing a <3 μm diameter circular laser line-scan (~100 to 154 mW at 800 nm) (*SI Appendix*, Fig. S1*A*) directly on the vessel lumen as visualized by the perfusion of intravenous (i.v.) dye (70 kDa FITC-dextran) (Fig. 1*A*). Laser power was applied in 20 second (s) increments for a total of 20 to 80 s until there was indication of halting blood flow and dye leakage (Fig. 1 *A* and *B*). Sham injuries were performed in separate vascular networks using identical laser powers (*SI Appendix*, Fig. S1*A*) localized next to similarly sized capillary segments (*SI Appendix*, Fig. S1*B*) without damaging the vessel (Fig. 1*B* and *SI Appendix*, Fig. S1*C*). Capillary diameter did not influence the laser power or time needed to rupture the vessel (*SI Appendix*, Fig. S1 *D* and *E*). Although, an increase in laser power, but not irradiation time, was needed for injuries deeper into the cortex (*SI Appendix*, Fig. S1 *F* and *G*).

To understand the consequence of focal capillary injury, injured regions were imaged longitudinally for 21 days (d). One to three days postinjury (dpi), the majority of vessels remained disconnected and lacked blood flow (Fig. 1*B* and *SI Appendix*, Fig. S1 *H* and *I*). Quantification of vessel length revealed that most vessels (59% of injuries) had completely receded back to their branch points 7 to 14 dpi (Fig. 1*C*). We defined these as regression events. A smaller portion of injured vessels (41%) reconnected and reestablished blood flow 7 dpi (Fig. 1*C* and *SI Appendix*, Fig. S1 *H* and *I*). We called these repair events. Sham injuries did not change the vessel segment length (Fig. 1*C*). The occurrence of regression or repair events did not appear to be due to differences in laser power, time, diameter, or vessel length (*SI Appendix*, Fig. S2 *A*–*D*). No consistent trend in capillary response was seen with vascular branch order (*SI Appendix*, Fig. S2*E*), but superficial capillaries (0 to 50 μm cortical depth) were more likely to regress (*SI Appendix*, Fig. S2*F*) even though lower powers were needed to induce injuries (*SI Appendix*, Fig. S2*G*). Overall, these findings show that precise laser line-scans can be used to induce focal capillary injury in vivo, with roughly ⅗ of injured vessels experiencing regression and ⅖ undergoing gradual repair.

Capillaries may appear to regress if the lumen collapses. We therefore examined whether capillaries that appeared to regress by visualization of i.v. dye involved endothelial loss. AAV-BR1-GFP was administered retro-orbitally to express GFP in the endothelium of a mural cell reporter mouse with a cranial window (27). Following a 2-wk incubation period, capillary injuries were performed. In three separate experiments, we found that the endothelium of injured capillaries indeed receded to their nearest branchpoints by 14 dpi (Fig. 1*D*). This confirmed that capillary regression events were most likely a complete loss of the respective capillary segment.

### Pericyte Death May Be Induced by Capillary Injury, but Remodeling of Neighboring Pericytes Ensures Coverage.

Following capillary injuries, death of nearby pericytes was occasionally observed 3 dpi (22% of injuries), leaving a portion of the capillary network transiently uncovered by pericytes (*SI Appendix*, Fig. S3*A*). Pericyte loss occurred occasionally when their processes were either directly injured by the line-scan, or indirectly in the absence of laser injury, presumably by exposure to leaked blood or alterations in blood flow. We did not observe pericytes migrating away from the injury site and all neighboring pericyte somas remained stable within their positions, suggesting that pericyte loss was most likely due to cell death (*SI Appendix*, Fig. S4). In contrast, pericyte loss was not observed following sham injuries, suggesting that local thermal damage to the parenchymal tissue does not drive pericyte loss.

Pericyte death triggered remodeling of neighboring pericytes, which extended their processes into areas of the endothelium lacking pericyte coverage (28, 29) (*SI Appendix*, Fig. S3*B*). Pericyte process growth was evident at 3 dpi, and in most cases, remodeling was complete by 14 dpi. In sham injuries, pericyte process territory negotiation was observed, evidenced by small local fluctuations in process length, which are known to occur in the healthy mouse brain (28, 29) (*SI Appendix*, Fig. S3*C*). Remodeling pericyte processes achieved an average extension of 62.67 μm, compared to 8.93 μm for pericytes at sham sites (*SI Appendix*, Fig. S3*D*). Analysis of uncovered vascular length following pericyte death confirmed that most vessels lacking pericyte contact were recovered by 7 to 14 dpi (*SI Appendix*, Fig. S3*E*).

As shown in past studies involving direct ablation of pericytes via their somata (28, 29), loss of pericyte coverage with capillary injury resulted in dilation of capillaries (*SI Appendix*, Fig. S3*F*). On average, uncovered vessels dilated to 117.5% of baseline, and returned approximately back to their basal diameters following recoverage by pericyte remodeling (*SI Appendix*, Fig. S3*G*). No significant vessel diameter changes were observed at sham injury sites. Thus, laser-induced capillary injury leads to loss of pericyte coverage in approximately ⅕ of cases, but remodeling of neighboring cells ensures that pericyte coverage is regained over days/weeks.

### Focal Capillary Injury Induces a Local Inflammatory Response.

**Fig. 2.**
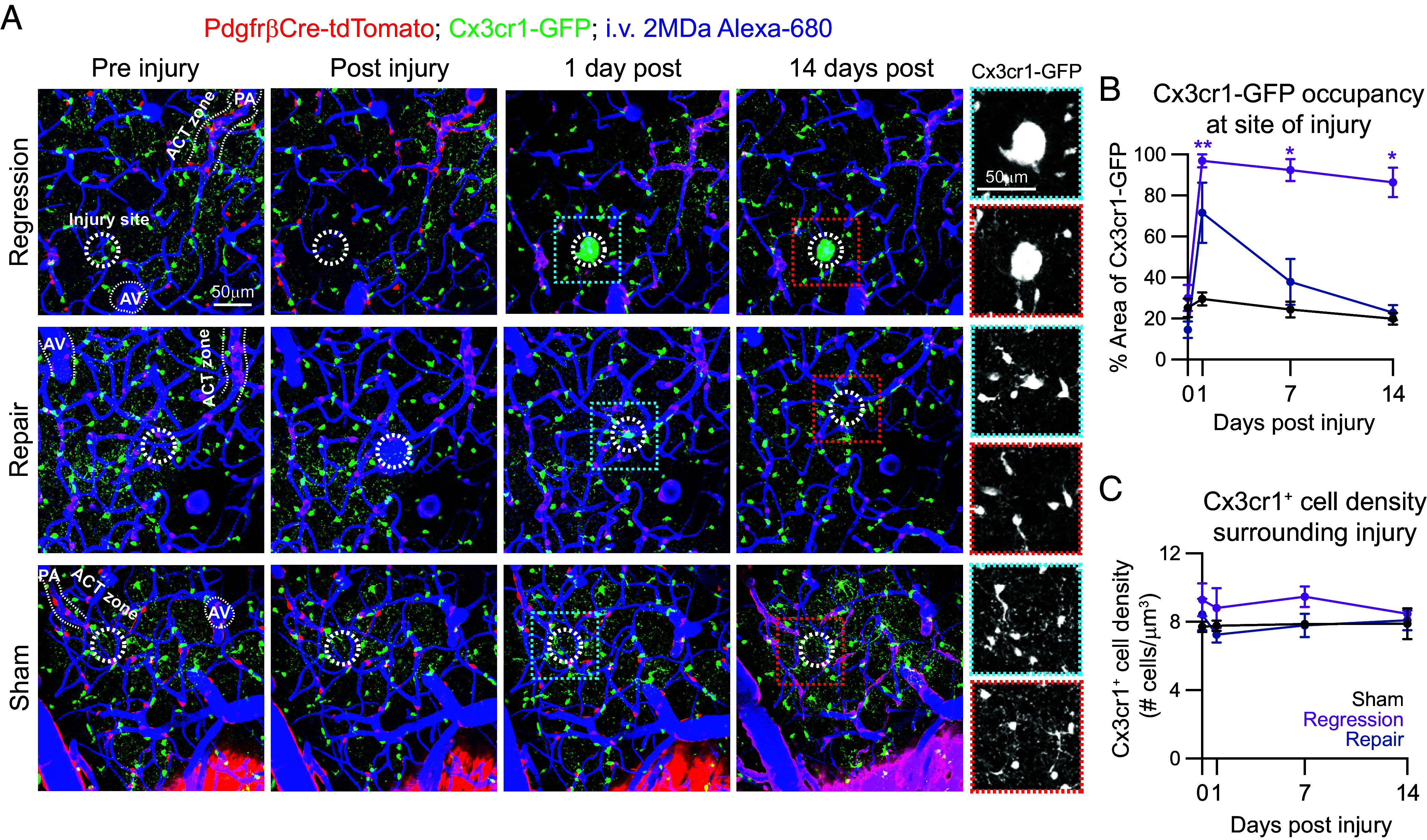
Focal capillary injury induces a local inflammatory response. (*A*) Representative in vivo images of a capillary injury in a PdgfrβCre-tdTomato; Cx3cr1-GFP mouse pre, post (~10 min), 1-, and 14-dpi. Pericytes are shown in red, Cx3cr1^+^ microglia and macrophages in green, and i.v. dye (2 MDa Alexa 680-Dextran) labeling vessels depicted in blue. Microvascular zones such as PA, ACT, and AVs are shown to depict the highly focal nature of the laser-induced capillary injuries and resulting neuroinflammatory reaction (25 μm radius; white dashed circle). *Insets* of these regions are shown in grayscale (1 d post blue outline and 14 d post red outline) indicating a possible mixture of soma and processes of Cx3cr1-GFP^+^ cells at the injury site. There may also be some autofluorescence occurring at the injury site in the regression example due to the robust inflammatory processes. Note: For the preinjury image of the regression event, the red and green channels were imaged separately from the far-red channel (shown in blue), within 5 min of each other, however there was a slight shift in the imaging frame resulting in an imperfect overlay of channels for that time point. (*B*) Graph of percent area of Cx3cr1-GFP^+^ cells at injury site preinjury (0 d) and 1-, 7-, and 14-dpi. ANOVA followed by Dunnett’s multiple comparison test were performed: Regression event: 0 vs. 1 d **P = 0.0074, 0 vs. 7 d: *P = 0.0109, 0 vs. 14 d: *P = 0.0385. Sham injuries n = 3, regression events n = 3, repair events n = 3; 2 mice. Data are shown as mean ± SEM. (*C*) Graph of Cx3cr1-GFP^+^ cell density surrounding injury site (excludes injury site) preinjury (0 d) and 1-, 7-, and 14-dpi. ANOVA tests were performed and no significant differences in microglia density were detected. Data are shown as mean ± SEM.

To assess the focality of the capillary injuries, we utilized PdgfrβCre-tdTomato; Cx3cr1-GFP mice to observe Cx3cr1-expressing microglia and macrophages following injury. Cx3cr1^+^ cells rapidly migrated to the injury site 1 dpi, which were presumably microglia from the surrounding parenchyma and vasculature (30) (Fig. 2 *A* and *B*). In regression events, Cx3cr1^+^ cells at the injury site remained significantly high 7 and 14 dpi. In contrast, Cx3cr1^+^ cells at the injury site decreased to baseline 14 dpi in repair events. Sham injuries did not result in a robust or prolonged inflammatory response at the site of laser ablation (Fig. 2 *A* and *B*). Outside of the irradiation site, we observed no change in Cx3cr1^+^ cell density following regression, repair, or sham events (Fig. 2*C*). This demonstrates that laser-induced capillary injuries initiate a highly focal inflammatory response that is prolonged in the event of vessel regression, but does not induce inflammatory conditions along other regions of the broader vascular network.

### Constriction of the Arteriole-Capillary Transition Zone Following Capillary Regression.

**Fig. 3.**
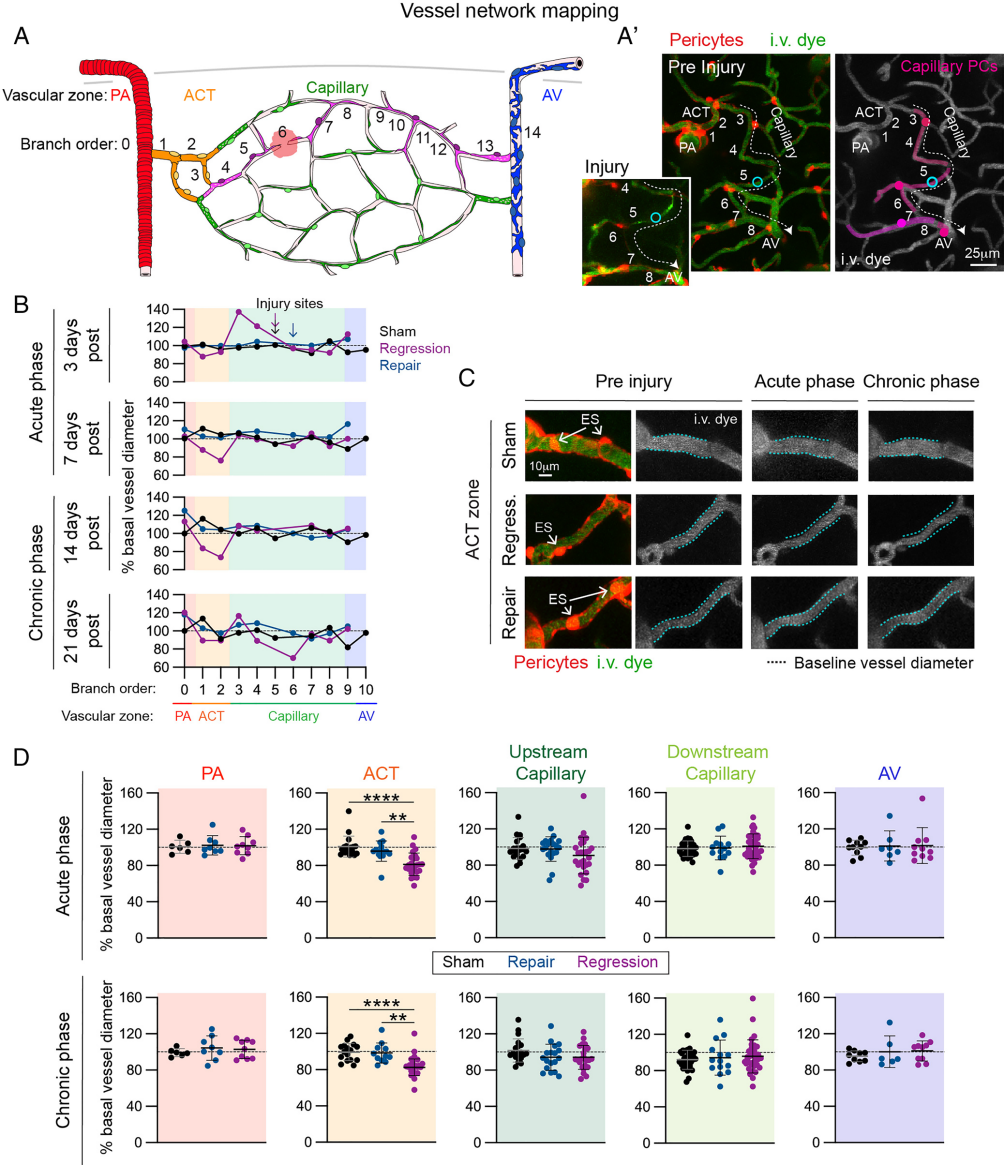
Chronic constriction of arteriole-capillary transition vessels occurs following laser-induced capillary regression. (*A*) Schematic of vessel network mapping by tracing pericyte territories (pink) and branch order from PA and arteriole-capillary transition (ACT) zone through the capillary network including the injury site to the AV. (*A’*) Representative in vivo image of a capillary injury with pericyte territories in a PdgfrβCre-tdTomato mouse preinjury with the line-scan path (cyan) and ~10 min postinjury (*Inset*). Pericytes are shown in red and i.v. dye (70 kDa FITC-Dextran) labeling vessels depicted in green. In the *Right* panel, pericyte territories are shown in pink, as well as vascular branch order and blood flow direction (dash line and arrow). (*B*) Graph of percent change in vessel diameter from baseline across the microvascular zones (PA, ACT, Capillary, AV) in a vessel network of a sham (black), regression (purple), and repair (blue) event. Changes in diameter are reported for each branch order in the acute phase (3 and 7 d) and the chronic phase (14 and 21 d). Branch order of injury sites in the capillary networks of the three case examples is denoted with color coded arrows. (*C*) Representative in vivo image of upstream ACT vessel segments from a sham, regression, and repair event preinjury and in the acute and chronic phase following capillary injury. Pericytes are shown in red and i.v. dye (70 kDa FITC-Dextran) labeling vessels depicted in green and grayscale. Soma of ensheathing pericytes are indicated with arrows. Baseline vessel diameter is indicated (dashed blue lines) to demonstrate ACT zone constriction following capillary regression. (*D*) Graphs of percent change in the diameter of vessel segments throughout the microvascular zones (PA, ACT, Capillary, AV) of sham (black), repair (blue), and regression (purple) events. Change from preinjury is shown during the acute (3 or 7 d) and chronic (14 or 21 d) phase following capillary injury. ANOVA followed by Tukey’s or Dunnett’s multiple comparison tests were performed depending on distribution of data. ACT zone: Acute: sham vs. regression ****P < 0.0001; repair vs. regression ***P = 0.0002. Chronic: sham vs. regression ***P = 0.0005; repair vs. regression **P = 0.0039. Each datapoint is the diameter from a single vessel segment. Sham = 12 experiments in 9 mice; repair = 8 experiments in 8 mice; regression = 14 experiments in 10 mice.

We next examined the effect of capillary injury on the broader microvascular network. For each experiment, we mapped the territory of morphologically distinct mural cells along the vascular tree to identify different vascular zones, including the PA, ACT zone, capillaries, and ascending venules (AVs) (Fig. 3*A*). We then measured the resting diameter of each vessel segment to understand whether focal capillary injury initiates a response in other vascular zones. We monitored diameter throughout two phases: 1) the acute phase, when the injured vessel was either reconnected (repaired) or undergoing regression (3 to 7 dpi), and 2) the chronic phase, when the regression had been completed and repaired vessels had been reconnected for at least a week (14 to 21 dpi). Strikingly, we found a specific decrease in vessel diameter in the upstream ACT zone following capillary regression during both the acute and chronic phases (Fig. 3 *B* and *C*). Some diameter changes were noted within the capillary zone with regression events, but no consistent pattern was maintained.

We found ACT zone constriction to be a consistent phenomenon following capillary regression in the acute and chronic phase (Fig. 3*D*). This pattern was observed in animals that underwent imaging experiments in either awake or anesthetized states (*SI Appendix*, Fig. S5). In the chronic phase, capillaries upstream from regressed vessels also exhibited decreased diameters in the anesthetized state, but this was not observed in the awake state (*SI Appendix*, Fig. S5).

Prominent constriction of the ACT zone was also seen as early as 10 min post capillary injury in awake, but not anesthetized, animals (*SI Appendix*, Fig. S6). Capillaries downstream of the injuries also exhibited a reduction in diameter 10 min post injury in both awake and anesthetized animals, likely due to loss of perfusion in those vessels (*SI Appendix*, Fig. S6). Overall, these data demonstrate that ACT zone constriction is a rapidly initiated, but lasting response irrespective of anesthetic state and occurs specifically with capillary regression after distal capillary injury.

### Constriction of the Arteriole-Capillary Transition Zone Is Independent of the Distance to the Capillary Injury Site and Inflammation Following Capillary Injury.

**Fig. 4.**
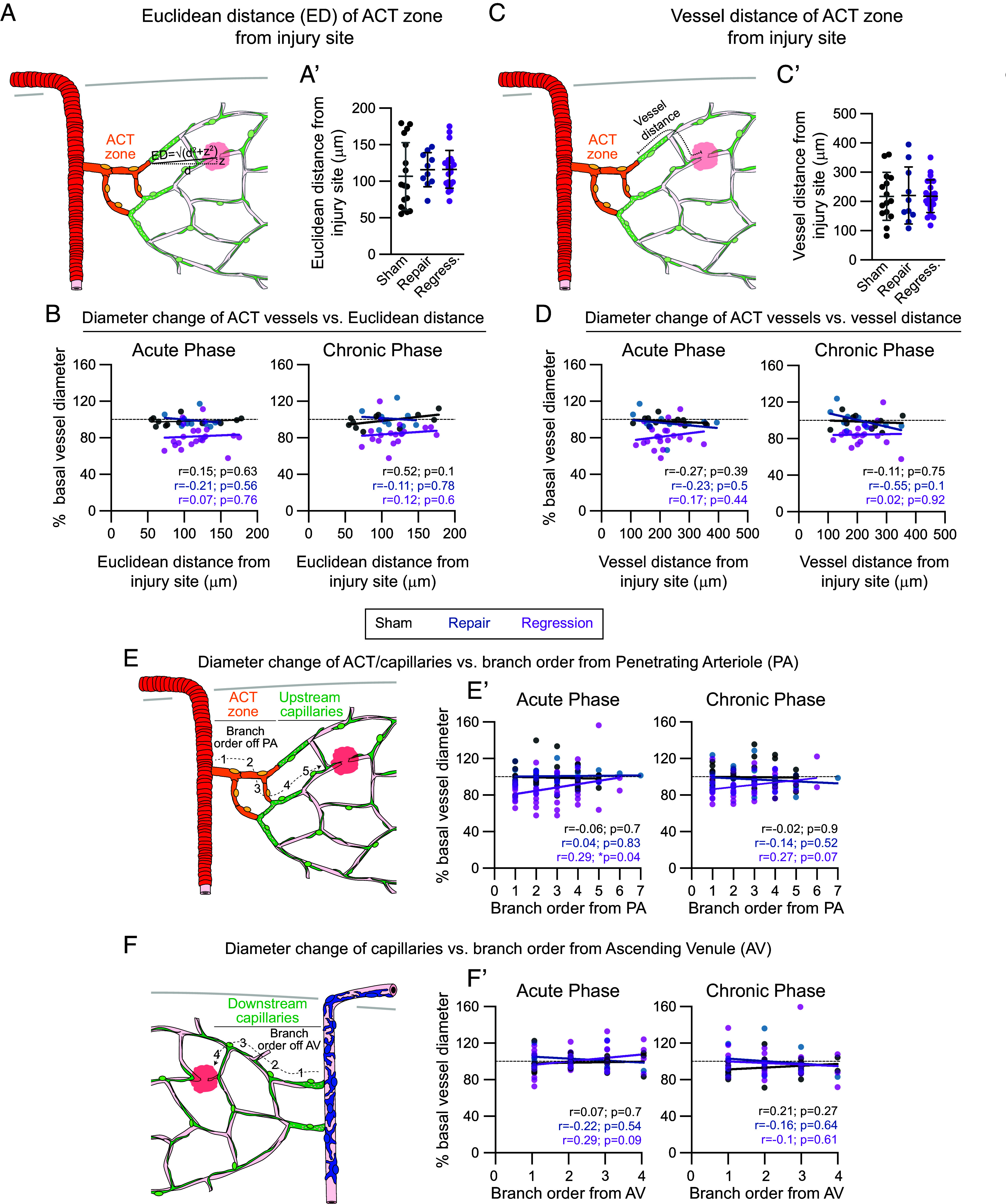
Proximity to the capillary injury site does not influence arteriole-capillary transition zone constriction. (*A*) Schematic showing Euclidean distance between arteriole-capillary transition (ACT) zone and capillary injury site. (*A’*) Euclidean distance between ACT zone and injury site in sham (black), repair (blue), and regression (purple) events. ANOVA indicated no significant difference. Sham = 12 experiments in 9 mice; repair = 8 experiments in 8 mice; regression = 14 experiments in 10 mice. (*B*) Scatter plots of Euclidean distance of ACT zone to capillary injury site versus ACT zone diameter change from baseline during the acute (3 or 7 d) and chronic (14 or 21 d) phases in sham, repair, and regression events. Pearson correlation tests did not show a correlation between diameter change and proximity to the injury site. Respective r and *P* values are indicated on all graphs for each experimental group. (*C*) Schematic showing vessel distance between ACT zone and capillary injury site. (*C’*) Shortest vessel distance between ACT zone and injury site between sham, repair, and regression events. ANOVA indicated no significant difference. (*D*) Scatter plots of vessel distance versus ACT zone diameter change from baseline during the acute (3 or 7 d) and chronic (14 or 21 d) phases in sham, repair, and regression events. Pearson correlation tests did not show a correlation between diameter change and proximity to the injury site. (*E*) Schematic showing capillary branch order from PA with (*E’*) scatter plots of vessel diameter changes at each branch order during the acute (3 or 7 d) and chronic (14 or 21 d) phases in sham, repair, and regression events. Pearson correlation indicated a significant correlation between diameter change in upstream vessels and proximity to the PA. (*F*) Schematic showing capillary branch order from AVs with (*F’*) scatter plots of vessel diameter changes at each branch order during the acute (3 or 7 d) and chronic (14 or 21 d) phases in sham, repair, and regression events. Pearson correlation tests did not show a correlation between diameter change in downstream vessels and proximity to the injury site.

We next examined whether the likelihood of ACT zone constriction was related to its proximity to the injury site, and thus potential exposure to leaked blood components or inflammation surrounding the injury site. The Euclidean and vessel distances between the ACT zone and injury site were comparable between sham, repair, and regression events (Fig. 4 *A* and *C*). Furthermore, we did not observe a relationship between the degree of ACT zone constriction with either Euclidean or vessel distance in the acute and chronic phases (Fig. 4 *B* and *D*). This demonstrates that proximity to the injury site does not play a role in ACT zone constriction.

We also examined whether activation of Cx3cr1^+^ microglia occurred around the ACT zone to determine whether inflammation could be inducing ACT zone constriction following capillary regression. We found no overt difference in how microglia interacted with the ACT zone via their processes or soma following sham, repair, or regression events (30) (*SI Appendix*, Fig. S7*A*). To determine whether microglia take on an ameboid morphology indicative of inflammation, Sholl analyses were performed on microglia surrounding the ACT zone in sham, repair, and regression experiments (31) (*SI Appendix*, Fig. S7*B*). Microglia maintained a consistent elaboration of processes extending from their soma (*SI Appendix*, Fig. S7*C*) as well as total process length and number of processes (*SI Appendix*, Fig. S7*D*) in sham, repair, and regression events at 3 and 14 dpi. This contrasts with microglia surrounding the injury site 1 dpi (*SI Appendix*, Fig. S7*E*) which showed a decrease in the elaboration of their processes from their soma (*SI Appendix*, Fig. S7*F*) as well as total process length and number of processes (*SI Appendix*, Fig. S7*G*). These data indicate that constriction of the ACT zone in regression events is not likely caused by injury and inflammatory processes elicited by microglia around the ACT zone.

To determine whether there was directionality in vasoconstriction toward upstream or downstream vessels centered around capillary regressions, we performed a deeper analysis of all vessels within each vessel network. We differentiated vessel segments based on their branch order from PAs or AVs for vessels upstream (Fig. 4*E*) or downstream (Fig. 4*F*) of the injury site, respectively. During the acute phase, there was a significant correlation driven by the constriction of vessels proximal to the PA only during regression events (Fig. 4*E’*). This trend was also observed in the chronic phase. No relationship regarding the degree of vessel constriction and proximity to the AVs was observed on the downstream side of the network during the acute and chronic phases in sham, repair, or regression events (Fig. 4*F’*). These data, along with the prominent constriction occurring within the ACT zone 10 min following a capillary injury in the awake state (*SI Appendix*, Fig. S6), suggest that upstream vessels have more prominent vasoconstriction from injured, regressing capillary networks.

### Vasomotor Dynamics in the Arteriole-Capillary Transition Zone Are Attenuated Following Capillary Injury.

**Fig. 5.**
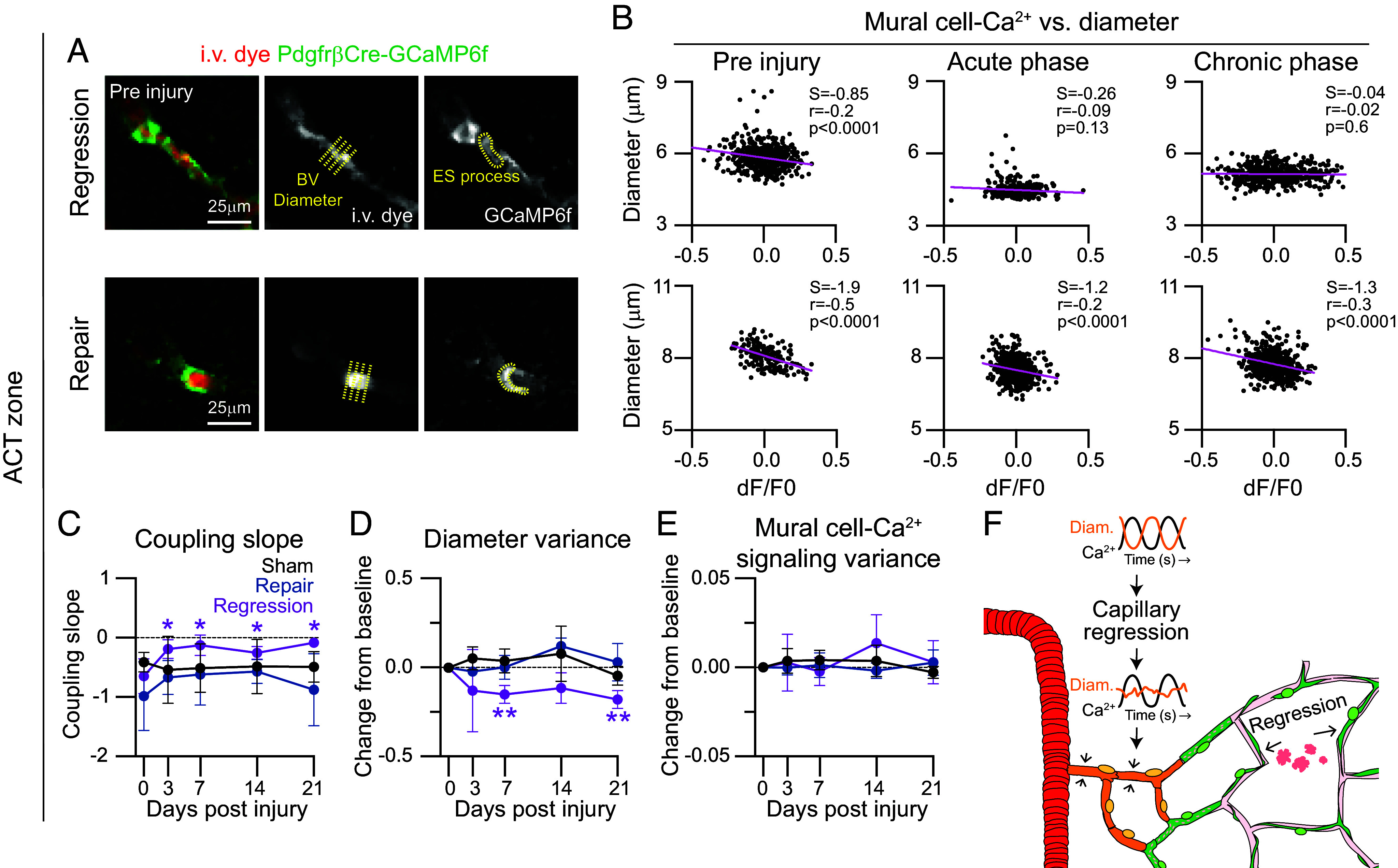
Vasomotor dynamics along the arteriole-capillary transition zone are attenuated following capillary regression. (*A*) Representative image of arteriole-capillary transition (ACT) zones from a regression and repair event in an awake PdgfrβCre-GCaMP6f mouse. Pericytes are shown in green and i.v. dye (70 kDa Texas Red-Dextran) labeling vessels depicted in red. Grayscale images show location of ACT diameter sampling (yellow crosslines) and region of interest (ROI, yellow outline) to measure GCaMP6 fluorescence intensity in ensheathing pericyte. (*B*) Scatter plots of change in mural cell GCaMP6f signal (dF/F0) versus ACT zone diameter over at least 2 min (data point collected every 0.512 s or 1.951 Hz) preinjury, acute (3 or 7 dpi) and chronic (14 or 21 dpi). Plots are shown following cross-correlation analysis (*SI Appendix*, Fig. S8*B*) with strongest correlation time shift shown. Regression correlation time: Pre t = 2.048 s, Acute t = 1.536 s, Chronic t = 1.024 s; Repair correlation time: Pre t = 1.024 s, Acute t = 1.024 s, Chronic t = 1.536 s. Pearson correlation tests were performed, and respective r and *P* values are reported on graphs along with the coupling slope (S). (*C*–*E*) Graphs of (*C*) coupling slope, (*D*) diameter variance, and (*E*) mural cell Ca^2+^ signal variance over the course of 21 d following injury in ACT zones upstream of sham (black), repair (blue), and regression (purple) events. ANOVA followed by Dunnett’s multiple comparison tests were performed: Coupling slope: Regression: 0 vs. 3 d: *P = 0.015, 0 vs. 7 d: *P = 0.027, 0 vs. 14 d: *P = 0.022, 0 vs. 21 d: *P = 0.038. Diameter variance: ACT zone–Regression: 0 vs. 7 d: **P = 0.002, 0 vs. 21 d: **P = 0.003. Sham n = 4, repair n = 3, regression n = 7; 5 mice. (*F*) Schematic demonstrating that capillary regression results in attenuated vasomotion (orange) with normal mural cell Ca^2+^ signaling (black) in the upstream ACT zone.

Vessel diameter in the ACT is dynamic, even during resting periods, and is generally inversely regulated by mural cell Ca^2+^ signaling fluctuations that influence the contractile and dilatory state of these vessels (15, 24). Thus, we next investigated whether resting vasomotion and mural cell Ca^2+^ dynamics were altered in the ACT zone following capillary regression. In vivo imaging was performed in awake PdgfrβCre-GCaMP6f mice, which express a genetically encoded calcium sensor in mural cells. By measuring the lumen diameter and GCaMP6f fluorescence intensity of mural cells in the ACT zone (Fig. 5*A* and *SI Appendix*, Fig. S8*A*) and PA (*SI Appendix*, Fig. S9 *A* and *B*) upstream of capillary injuries, we were able to study the dynamics and relationship between mural cell Ca^2+^ and vasomotion during periods of rest. Cross-correlation analyses were conducted to identify the timing shift needed for the strongest correlation between mural cell Ca^2+^ and vessel diameter, since Ca^2+^ changes should precede diameter changes. The linear slope, i.e., “coupling slope,” allowed us to determine the strength of the inverse relationship between mural cell Ca^2+^ and vessel diameter (*SI Appendix*, Figs. S8*B* and S9*C*). Diameter changes generally followed fluctuations in mural cell Ca^2+^ with a delay of 1 to 2 s for both the ACT zone and PAs. Following capillary regression, we found a weaker coupling slope in the ACT zone during the acute and chronic phases (Fig. 5 *B* and *C*) but not the PA (*SI Appendix*, Fig. S9*D*). In contrast, sham and repair events maintained strong coupling along ACT vessels and PAs throughout the time course (Fig. 5*C* and *SI Appendix*, Fig. S10*A*). The negative correlation typically observed between mural cell Ca^2+^ and diameter at the ACT zone diminished on day 3 and 7 following capillary regression (*SI Appendix*, Fig. S11*C*); however, this was not observed with PAs (*SI Appendix*, Fig. S11*A*). Further, we did not find any significant changes in coupling time for PAs (*SI Appendix*, Fig. S11*B*) and ACT zones (*SI Appendix*, Fig. S11*D*) indicating there is likely no effect in the physiological timing of mural cell Ca^2+^ and diameter following sham, repair, and regression events.

Interestingly, we found an attenuation of diameter variance (Fig. 5*D* and *SI Appendix*, Fig. S8*C*), but not mural cell Ca^2+^ signaling variance (Fig. 5*E* and *SI Appendix*, Fig. S8*D*), in the ACT zone upstream of regressed capillaries. Within the vasomotor frequency range of 0.025 to 0.2 Hz (32), the power of mural cell Ca^2+^ oscillations remained unchanged, while the power of vessel diameter oscillations was reduced (*SI Appendix*, Fig. S12 *A* and *B*). However, the mean vasomotor frequency of ~0.1 Hz was maintained in both the diameter and mural cell Ca^2+^ oscillations (*SI Appendix*, Fig. S12 *C* and *D*).

Relatively normal vascular diameter and mural cell Ca^2+^ variance was maintained along PAs following sham, repair, and regression events (*SI Appendix*, Figs. S9 *E* and *F* and S10 *B* and *C*). Altogether, these data highlight a chronic attenuation of ACT zone vasodynamics following capillary regression (Fig. 5*F*).

### Constriction of the Arteriole-Capillary Transition Zone Results in Blood Flow Deficits.

**Fig. 6.**
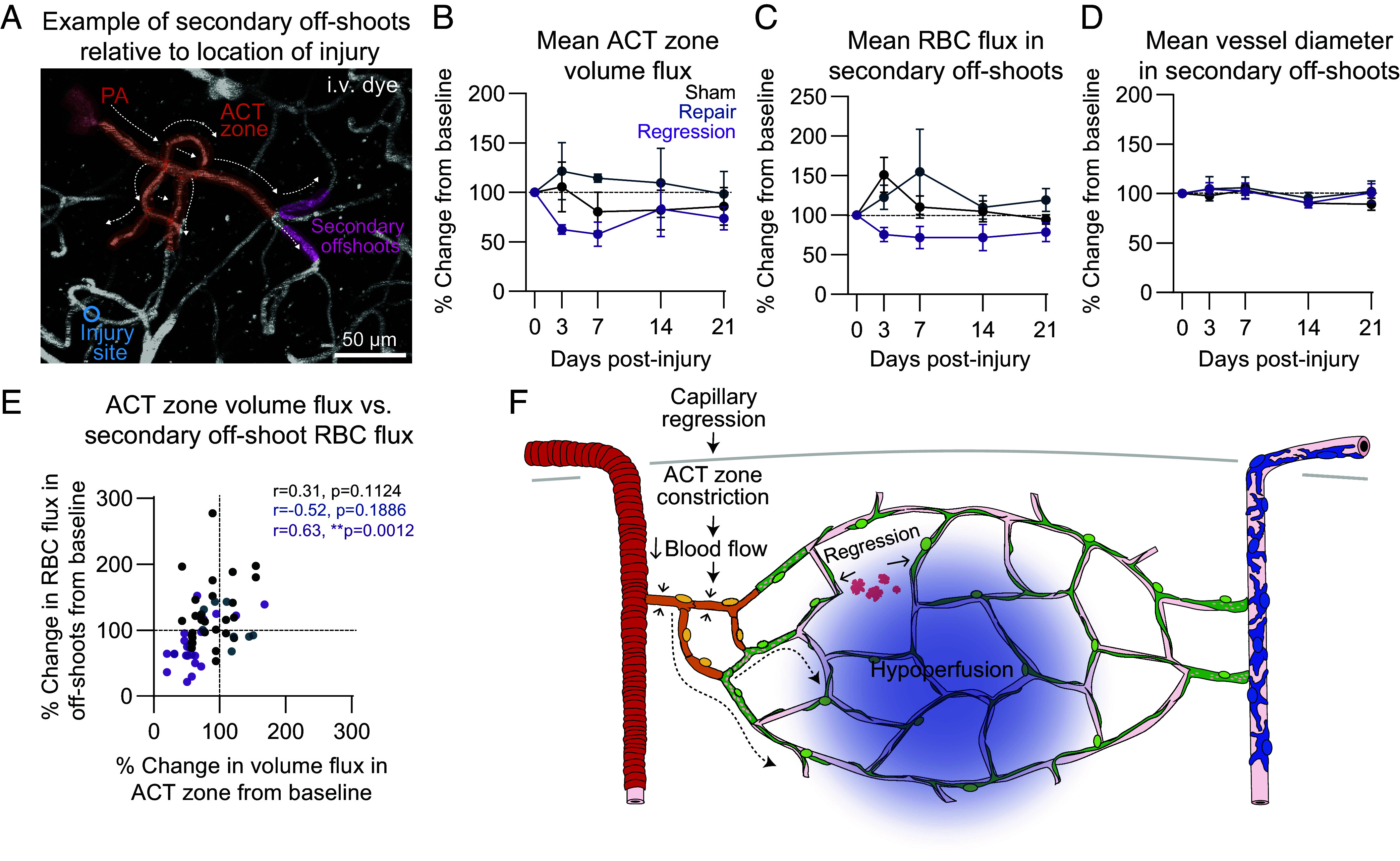
Capillary regression and chronic constriction of the ACT zone results in hypoperfusion of secondary off-shoot vessels. (*A*) Image and schematic showing location of secondary off-shoots relative to injury site. (*B*) Graph of upstream ACT zone blood volume flux over the course of 21 d following sham, repair, and regression events in awake mice. A mixed-effects model was performed: Time: *P* = 0.6531, Condition: *P* = 0.1137, Interaction: *P* = 0.6922. Sham *n* = 3 vessels (3 mice), repair *n* = 2 vessels (2 mice), regression *n* = 6 vessels (2 mice). Data are shown as mean ± SEM. (*C*) Graph of red blood cell flux over the course of 21 d in secondary off-shoots from ACT zone following sham (black), repair (blue), and regression (purple) events in awake mice. A mixed-effects model followed by Tukey’s multiple comparisons test was performed: Time: *P* = 0.2798, Condition: *P = 0.0329, *Interaction: *P* = 0.0356. Sham vs. regression day 3: *P = 0.0243. Sham n = 9 vessels (3 mice), repair n = 3 vessels (3 mice), regression n = 7 vessels (3 mice). Data are shown as mean ± SEM. (*D*) Graph of vessel diameter over the course of 21 d following injury in secondary off-shoots from ACT zone following sham, repair, and regression events in awake mice. A mixed-effects model was performed: Time: *P* = 0.0809, Condition: *P* = 0.6315, Interaction: *P* = 0.8773. Sham n = 9 vessels (3 mice), repair n = 3 vessels (3 mice), regression n = 7 vessels (3 mice). Data are shown as mean ± SEM. (*E*) Scatter plot of ACT zone blood volume flux versus RBC flux in secondary off-shoots over 21 dpi in sham, repair, and regression events in awake mice. Pearson correlation tests were performed, respective r and *P* values are reported on graph. (*F*) Schematic demonstrating that capillary regression results in ACT zone constriction reducing blood flow into the microvascular network including uninjured, secondary off-shoots.

To understand the effect of ACT zone constriction on blood flow, we used low power line-scans in awake animals to track blood cell movement in both the ACT zone and secondary off-shoots from a different branch of the ACT zone than the injury (Fig. 6*A*). In the event of a capillary regression, blood volume flux in the ACT zone was reduced to ~55 to 85% of basal levels, generally to greater degrees than sham and repair events (Fig. 6*B*). Blood cell flux in the secondary off-shoots was reduced to ~75% of basal levels in regression events for at least 21 d (Fig. 6*C*). This trend was not observed in secondary off-shoots in sham or repair events. Importantly, lumen diameter was unchanged in the secondary off-shoots, indicating that flow deficits were not due to local capillary constriction (Fig. 6*D*). Rather, the reduction in blood flow in the secondary off-shoots significantly correlated with the reduction in volume flux in the ACT zone (Fig. 6*E*). Thus, ACT zone constriction following capillary regression reduces blood flow into the downstream capillary bed (Fig. 6*F*).

## Discussion

While the conductive nature of the brain microvasculature is involved in normal blood flow regulation, this trait may become a source of vulnerability in neurological conditions involving capillary injury. We show that injury and regression of single capillaries causes chronic and selective vasoconstriction in the upstream ACT zone, which can be located hundreds of micrometers away but supplies blood to the broader capillary network. This chronic vasoconstriction reduces blood flow to ~55 to 85% of basal levels in the ACT zone and ~75% of basal levels in downstream secondary capillaries, creating an unexpected link between capillary regression and local hypoperfusion. Capillary regression attenuates the degree of vasomotion in the ACT zone where mural cell Ca^2+^ dynamics are preserved but fail to translate into lumen diameter changes. Our experiments ruled out inflammation and proximity to the injury site as potential factors in ACT zone vasoconstriction. However, aberrant upstream conductance of endothelial signals remains a potential explanation, and future mechanistic studies will be essential to test this possibility.

Prior studies have shown that the ACT zone, along with the precapillary sphincter just upstream in select arterioles, can dilate rapidly to promote blood flow during functional hyperemia (20–23), or potently constrict to mediators such as endothelin-1 and ATP (33). Relating our findings to models of neurological disease, sustained vasoconstriction has been reported after reperfusion with both focal and global cerebral ischemia (24, 34). In these prior studies, the location of constriction was most prominent in the ACT zone. Further, vasoconstriction in the ACT zone is observed with potassium-induced cortical spreading depolarization (35), and this may contribute to lasting blood flow deficits. Note that the ACT zone has also been referred to as “precapillary arterioles” or “first/second-order capillaries,” but reports of its unique sensitivity seem to be consistent despite differing nomenclature.

The degree of vasomotion was significantly reduced in the ACT zone following capillary regression. However, GCaMP6 signal variance in mural cells was unaffected. This translated to weakened coupling slopes throughout acute and chronic time frames. One possible explanation for this is uncoupling of mural cell Ca^2+^ from vasomotor dynamics. This could be due to alterations in Ca^2+^-dependent pathways that stimulate actomyosin cross-bridging (calmodulin and myosin light chain kinase), alpha-smooth muscle actin and myosin, or other cytoskeletal elements that facilitate contractility (36). While uncoupling remains a possibility, the consistency in vasomotor oscillations following capillary regressions would suggest that an unknown factor is eliciting a more permanent vasoconstrictive state and overriding mural cell Ca^2+^ control of vessel diameter. This could be due to aberrant contraction of ensheathing pericytes through Ca^2+^-independent mechanisms. Rho kinase signaling promotes mural cell contraction by inhibiting myosin light chain phosphatase through rho-GTP. The upregulation of this pathway during ischemic injury and subarachnoid hemorrhage is well known (37) and rho kinase inhibition has been shown to improve blood flow by attenuating cerebral vasospasm (38). Another candidate is endothelin-1, which is a potent vasoconstrictor up-regulated in hemorrhagic stroke, Alzheimer’s Disease (AD), and multiple sclerosis (39). It acts through the G-coupled protein receptor, endothelin receptor Type-A, to induce mural cell contraction through Ca^2+^-dependent and independent mechanisms (40–42). Finally, there may be a physical change in the vessel wall that restricts vasoreactivity, such as thickening in the vascular basement membrane (43, 44). The mechanism/s underlying chronic vasoconstriction and disruption of vasodynamics in the ACT zone, whether through uncoupling or vasoconstricting factors, warrants further investigation.

We observed a difference in outcome between capillary injuries that involved regression or repair of the targeted capillary, even though similar laser irradiation parameters were used (*SI Appendix*, Fig. S2). The severity of injury was comparable between groups, as both exhibited initial microbleeds followed by hyperacute constriction within 10 min of injury (*SI Appendix*, Fig. S6). Acute ACT constriction may be an initial response to limit leakage and bleeding following capillary rupture. However, only those involving capillary regression were associated with chronic ACT zone vasoconstriction and reduced vasomotor dynamics. It is conceivable that loss of capillary connectivity creates lasting depression or disorganization in endothelial membrane voltage and Ca^2+^ signaling, which reduces input to the ACT zone. Indeed, recent imaging studies have shown complex local Ca^2+^ signals within the endothelium, which are potentiated by conductive hyperpolarizing signals (45). These findings suggest that repair of the injured capillary segment, and potentially reestablishment of appropriate conductive responses, is a means to alleviate ACT zone constriction.

Capillary regression is a common event in normal aging, AD, VCID, and many other progressive neurological diseases (9, 10, 46). In aged animals, the ACT zone and precapillary sphincter exhibit impaired neurovascular coupling responses (47). This has been attributed to poor responsiveness to vasomodulators. Our data suggest that there may be an additional link to capillary regression in the same vascular networks. In this regard, future studies are needed to determine whether capillary regression co-occurs with ACT zone constriction in aging and disease. Interestingly, a recent study in the aged mouse brain reported ACT *dilation* associated with age-related capillary density loss. However, cortical depth analysis revealed that deeper cortical layers exhibited more severe capillary rarefaction associated with ACT vasoconstriction (47). Further, deep two-photon imaging in awake mice showed age-related hypoperfusion was greatest in the callosal white matter (48). Thus, brain location may be key during studies to relate capillary regression with cerebral blood flow.

There are some limitations to these studies. First, it is unclear whether the optical ablation approach produces a form of vascular injury that is representative of disease pathology. For example, we observed a robust microglia response following capillary injury that may occur following a traumatic injury to the brain but is likely not representative of microglia responses during progressive pathology like aging. Therefore, other targeted approaches to induce capillary regression in a zone-specific manner will be instructive in determining whether the phenomenon is technique specific. Second, we did not apply our optical ablation approach to models with disease-relevant factors, and responses in these models may differ from healthy adult mice. Third, our studies are restricted to relatively superficial layers of the cortex (upper 200 μm) because optical ablations could only be effectively generated at these depths due to light scattering. Layer-specific differences in vascular vulnerability and architecture will need to be considered in future studies. Finally, our experiments with Cx3cr1-reporter animal numbers are low and so while our observations were consistent between animal and injury type, there remains a possibility that some of these studies are underpowered to detect small differences in responses by Cx3cr1-expressing cells.

In summary, our studies reveal how capillary-level pathology can produce outsized effects on perfusion by acting distally upon upstream ACT vessels. This link may be a contributing factor in the cerebral hypoperfusion detected in VCID, AD, and chronic phases of acute neurological injuries (stroke, hemorrhage, traumatic brain injury). Since the ACT zone is a crucial gate for blood delivery in the brain, it is imperative to understand how this vascular zone is affected in various disease conditions and whether it might be a therapeutic target for improvement of cerebral blood flow.

## Materials and Methods

### Animals.

Mice were housed in a specific pathogen-free facility approved by AAALAC and were handled in accordance with protocols approved by the Seattle Children’s Research Institute IACUC. PdgfrβCre-tdTomato and PdgfrβCre-GCaMP6f mice were created by breeding Pdgfrβ-Cre mice (FVB and C57BL/6 × 129 background) (49) with Ai14-flox (Jax #007914) (50) or Ai95-flox (Jax #028865) (51) mice (C57BL/6 backgrounds) to generate mural cell reporter lines. PdgfrβCre-tdTomato mice were crossed with Cx3cr1-GFP (Jax #005582) mice (C57BL/6 background) (52) to simultaneously label mural cells and microglia. Both male and female mice within 3 to 8 mo of age were utilized.

### Cranial Window Surgery and In Vivo Two-Photon Imaging.

Chronic, skull-removed cranial windows were placed over the somatosensory cortex for in vivo imaging, as previously described (28). Mice were allowed to recover for at least 3 wk prior to imaging. For vascular labeling, various fluorescent dextrans were injected retro-orbitally under deep isoflurane anesthesia (2% MAC in medical-grade air). PdgfrβCre-tdTomato mice were injected with 25 µL of 5% (w/v in saline) 70 kDa FITC-dextran (Sigma-Aldrich; 46945), PdgfrβCre-tdTomato; Cx3cr1-GFP mice were injected with 25 µL of 5% (w/v in saline) custom Alexa Fluor 680 (Life Technologies; A20008) conjugated to 2MDa Dextran (Fisher Scientific; NC1275021) (53), and PdgfrβCre-Ai95 mice were injected with 25 µL of 2.5% (w/v in saline) 70 kDa Texas Red™ dextran (Invitrogen™; D1864). During imaging of PdgfrβCre-tdTomato and PdgfrβCre-tdTomato; Cx3cr1-GFP mice, isoflurane was maintained at ~1.5% MAC in medical-grade air. For awake imaging experiments, PdgfrβCre-Ai95 mice were habituated to head fixation on a treadmill (PhenoSys speedbelt) allowing free forward-backward movement during a 1 to 2 h imaging session. For subsequent imaging experiments, mice were briefly anesthetized with isoflurane to retro-orbitally inject fluorescent dextran, and then allowed to wake up for at least 10 min prior to imaging. Imaging was performed with a Bruker Investigator coupled to a Spectra-Physics Insight X3. The laser was tuned for 975 nm excitation when imaging PdgfrβCre-tdTomato mice, and 920 nm excitation was used for PdgfrβCre-tdTomato; Cx3cr1-GFP and PdgfrβCre-Ai95 mice. Collection of green, red, and far-red fluorescence emission was achieved with 525/70, 595/50, and 660/40 emission bandpass filters respectively, and detected with Hamamatsu GaAsP photomultiplier tubes. A 20× (1.0 NA) water-immersion objective (Olympus; XLUMPLFLN) was used to collect high-resolution images. For structural imaging of the brain vasculature, z-stacks were collected at 1.0 μm z increments at 347 μm × 347 μm (512 × 512 pixel resolution) using 3.6 μs/pixel dwell time. For blood flow analysis, three trials of 1.3 s line-scans were collected along the ACT zone (1st and 2nd order vessels) and uninjured, secondary capillaries with at least 10 s lags in between each line-scan. For imaging of mural cell Ca^2+^ signaling and vasomotion in PdgfrβCre-Ai95 mice, movies were captured for 307.2 s at 1.951 Hz at 295 μm × 295 μm (256 × 256 pixel resolution) averaging every two frames using 1.2 μs/pixel dwell time.

### Capillary Injury Using Two-Photon Irradiation.

Capillary segments to target for injuries were chosen by identifying capillary segments both within the upper 200 μm of the cortex and greater than 4 branch orders from a PA. The majority of injuries were induced by creating a circular line-scan path (<3 μm in diameter) to apply ~100 to 154 mW of power at 800 nm excitation directly onto the vessel for 20 to 80 s in 20 s increments at 3.6 μs/pixel dwell time. Scanning was stopped when blood flow halted (stalled dye and/or red blood cells) and leakage of i.v. dye was observed. Sham injuries were performed in separate vascular networks using similar parameters focused on a parenchymal region adjacent to a capillary segment. Extended details on how capillary injuries were optimized and performed are provided with *SI Appendix*. Generally, multiple experiments were performed in each animal. Each experiment was performed in entirely separate regions perfused by different PAs. Usually, 2 to 3 experiments were performed along the same timeline in anesthetized mice. In experiments where the animals were awake, mostly one experiment was performed over a course of 3 wk to ensure all image/data types could be captured within a 2-h imaging limit with minimal animal movement.

### Quantification of the Microglia Response to Focal Capillary Injury.

Maximum projected images (347 μm × 347 μm × 50 μm) were created following experiments performed in PdgfrβCre-tdTomato; Cx3cr1-GFP mice. Images encompassed the injured vessel segment and connecting vascular zones throughout all time points. Following thresholding of the Cx3cr1-GFP channel, the GFP-positive area in the 25 μm radius (1,963.5 μm^2^) surrounding the injury site, which encompassed the initial Cx3cr1-GFP response, was measured in FIJI. The percentage of GFP-positive area occupying the 1,963.5 μm^2^ region of interest was calculated to identify the focal microglia response to capillary and sham injury. The number of Cx3cr1-GFP-positive microglia outside of the injury site in the respective images were counted using cell counter function in FIJI. Total tissue volume for each image was calculated and microglia density was normalized to µm^3^ of tissue.

### Topological Analysis of Vessel Diameter.

To analyze vessel diameter changes across the microvascular web following capillary injury and sham experiments, the territory of capillary pericytes along the vascular tree was mapped out using the SNT FIJI plugin to identify how each microvascular zone connects from PA to AV. Experiments in which pericytes died due to capillary injury were not included. Precapillary sphincters were not easily discerned in our datasets (region of vessel narrowing with distended bulb downstream) (20) and were therefore not separately assessed. Vessel diameter at each branch point was measured in FIJI using the VasoMetrics plugin on maximum projections of each vessel segment (54). Vessel segments were measured at each time point and the percent change was calculated based on preinjury vessel diameter.

### Analysis of Distance between the Injury Site and the ACT Zone.

To measure the Euclidean distance between the injury site and the ACT zone, the distance between the focal plane of the injury site and the focal plane of the ACT zone (z) was determined. Then, the lateral distance between the injury site and the ACT zone in the x-y plane (d) was measured using the line function in FIJI on the max projected image. The equation $\sqrt{z^{2}+d^{2}}$ was then used to calculate the Euclidean distance. To measure the vessel distance between the injury site and the ACT zone, the length of each vessel segment between the two sites was measured using the SNT FIJI plugin.

### Analysis of Vasomotion and Mural Cell Ca^2+^ Dynamics.

In awake PdgfrβCre-Ai95 mice, movies were collected of smooth muscle cells on PAs and ensheathing pericytes on the 1st and 2nd order ACT zone upstream of each injury. In contiguous movies, any imaging frames where the animal had moved and the imaging period of ~10 s after movement were excluded from analysis to only assess resting vasomotion. To analyze vasomotion, the VasoMetrics plugin was utilized to measure vessel diameter in each frame (54). To analyze mural cell Ca^2+^ signatures in FIJI, images were despeckled and a ROI was drawn around the smooth muscle cells and ensheathing pericyte processes. We always ensured the ROIs encompassed the regions we were measuring across all frames. In the event a major shift was observed, ROIs were redrawn for those portions of the movies to always maintain consistency in where the GCaMP6f signal was being measured. The fluorescent intensity profile throughout the time series data was obtained using the plot z-axis profile function and the change in fluorescence divided by the median fluorescence intensity (dF/F0) was calculated. Vessel diameter and dF/F0 were plotted against each other to analyze their relationship. The coupling slope was determined by performing correlation and linear regression analysis at each time point to determine the strongest correlation efficient (r) and slope (coupling slope), respectively. This also allowed us to determine the “coupling time,” or the lag time it took for the diameter to respond to fluctuations in mural cell Ca^2+^ signaling based on the strongest correlation and coupling slope. Raw data were used to calculate linear regression, cross-correlation, and variance.

### Blood Flow Analysis.

In awake PdgfrβCre-Ai95 mice, line-scans were collected of 1st- or 2nd-order ACT zone vessels upstream of the injury site, and of uninjured, secondary capillary off-shoots of a different branch of the ACT zone than the injured capillary. Following collection of blood flow images using line-scans, red blood cell (RBC) velocity was measured in the ACT zone using MATLAB code for line-scanning particle image velocimetry (55) and blood volume flux was calculated using F = ⅛(π)(velocity)(diameter)^2^. In the secondary capillary offshoots, RBC flux was measured by counting the number of RBC shadows in each line-scan using the CellCounter plugin in FIJI and normalizing the numbers to represent flux over a 1 s period. Blood flow measurements from the 3 line-scans were averaged for all blood flow measurements.

### Statistics.

All statistical analyses were performed in GraphPad Prism (ver. 9). Respective statistical analyses are reported in each figure legend. Normality tests, generally Shapiro–Wilk tests, were performed on necessary datasets prior to statistical tests. SD is reported in all graphs unless specified otherwise.

## Supplementary Material

Appendix 01 (PDF)

## Data Availability

All data included in the article and have been deposited here: https://osf.io/xzt6r/ (56).

## References

[1] S. J. van Veluw , Detection, risk factors, and functional consequences of cerebral microinfarcts. Lancet Neurol. **16**, 730–740 (2017), 10.1016/S1474-4422(17)30196-5. 28716371 PMC5861500

[2] S. M. Greenberg , Cerebral microbleeds: A guide to detection and interpretation. Lancet Neurol. **8**, 165–174 (2009), 10.1016/S1474-4422(09)70013-4. 19161908 PMC3414436

[3] P. M. Summers , Functional deficits induced by cortical microinfarcts. J. Cereb. Blood Flow Metab. **37**, 3599–3614 (2017), 10.1177/0271678X16685573. 28090802 PMC5669342

[4] D. R. Thal , Two types of sporadic cerebral amyloid angiopathy. J. Neuropathol. Exp. Neurol. **61**, 282–293 (2002), 10.1093/jnen/61.3.282. 11895043

[5] R. Nortley , Amyloid β oligomers constrict human capillaries in Alzheimer’s disease via signaling to pericytes. Science **365**, eaav9518 (2019), 10.1126/science.aav9518. 31221773 PMC6658218

[6] R. J. Turner, F. R. Sharp, Implications of MMP9 for blood brain barrier disruption and hemorrhagic transformation following ischemic stroke. Front. Cell Neurosci. **10**, 56 (2016), 10.3389/fncel.2016.00056. 26973468 PMC4777722

[7] R. G. Underly , Pericytes as inducers of rapid, matrix metalloproteinase-9-dependent capillary damage during ischemia. J. Neurosci. **37**, 129–140 (2017), 10.1523/JNEUROSCI.2891-16.2016. 28053036 PMC5214626

[8] M. Fisher, S. French, P. Ji, R. C. Kim, Cerebral microbleeds in the elderly: A pathological analysis. Stroke **41**, 2782–2785 (2010), 10.1161/STROKEAHA.110.593657. 21030702 PMC3079284

[9] B. Schager, C. E. Brown, Susceptibility to capillary plugging can predict brain region specific vessel loss with aging. J. Cereb. Blood Flow Metab. **40**, 2475–2490 (2020), 10.1177/0271678X19895245. 31903837 PMC7820682

[10] M. van Dinther , Assessment of microvascular rarefaction in human brain disorders using physiological magnetic resonance imaging. J. Cereb. Blood Flow Metab. **42**, 718–737 (2022), 10.1177/0271678X221076557. 35078344 PMC9014687

[11] P. Blinder , The cortical angiome: An interconnected vascular network with noncolumnar patterns of blood flow. Nat. Neurosci. **16**, 889–897 (2013), 10.1038/nn.3426. 23749145 PMC4141079

[12] N. Nishimura , Targeted insult to subsurface cortical blood vessels using ultrashort laser pulses: Three models of stroke. Nat. Methods **3**, 99–108 (2006), 10.1038/nmeth844. 16432519

[13] T. A. Longden , Capillary K+-sensing initiates retrograde hyperpolarization to increase local cerebral blood flow. Nat. Neurosci. **20**, 717–726 (2017), 10.1038/nn.4533. 28319610 PMC5404963

[14] P. Thakore , Brain endothelial cell TRPA1 channels initiate neurovascular coupling. Elife **10**, e63040 (2021), 10.7554/eLife.63040. 33635784 PMC7935492

[15] C. Glück , Distinct signatures of calcium activity in brain mural cells. Elife **10**, e70591 (2021), 10.7554/eLife.70591. 34227466 PMC8294852

[16] J. A. Filosa , Local potassium signaling couples neuronal activity to vasodilation in the brain. Nat. Neurosci. **9**, 1397–1403 (2006), 10.1038/nn1779. 17013381

[17] D. A. Hartmann, V. Coelho-Santos, A. Y. Shih, Pericyte control of blood flow across microvascular zones in the central nervous system. Annu. Rev. Physiol. **84**, 331–354 (2022), 10.1146/annurev-physiol-061121-040127. 34672718 PMC10480047

[18] T. Kovacs-Oller, E. Ivanova, P. Bianchimano, B. T. Sagdullaev, The pericyte connectome: Spatial precision of neurovascular coupling is driven by selective connectivity maps of pericytes and endothelial cells and is disrupted in diabetes. Cell Discov. **6**, 39 (2020), 10.1038/s41421-020-0180-0. 32566247 PMC7296038

[19] R. I. Grant , Organizational hierarchy and structural diversity of microvascular pericytes in adult mouse cortex. J. Cereb. Blood Flow Metab. **39**, 411–425 (2019), 10.1177/0271678X17732229. 28933255 PMC6399730

[20] S. Grubb , Precapillary sphincters maintain perfusion in the cerebral cortex. Nat. Commun. **11**, 395 (2020), 10.1038/s41467-020-14330-z. 31959752 PMC6971292

[21] S. A. Zambach , Precapillary sphincters and pericytes at first-order capillaries as key regulators for brain capillary perfusion. Proc. Natl. Acad. Sci. U.S.A. **118**, e2023749118 (2021), 10.1073/pnas.2023749118. 34155102 PMC8255959

[22] C. N. Hall , Capillary pericytes regulate cerebral blood flow in health and disease. Nature **508**, 55–60 (2014), 10.1038/nature13165. 24670647 PMC3976267

[23] R. L. Rungta, E. Chaigneau, B. F. Osmanski, S. Charpak, Vascular compartmentalization of functional hyperemia from the synapse to the pia. Neuron **99**, 362–375.e4 (2018), 10.1016/j.neuron.2018.06.012. 29937277 PMC6069674

[24] R. A. Hill , Regional blood flow in the normal and ischemic brain is controlled by arteriolar smooth muscle cell contractility and not by capillary pericytes. Neuron **87**, 95–110 (2015), 10.1016/j.neuron.2015.06.001. 26119027 PMC4487786

[25] D. A. Hartmann , Pericyte structure and distribution in the cerebral cortex revealed by high-resolution imaging of transgenic mice. Neurophotonics **2**, 041402 (2015), 10.1117/1.NPh.2.4.041402. 26158016 PMC4478963

[26] S. Taylor , Suppressing interferon-γ stimulates microglial responses and repair of microbleeds in the diabetic brain. J. Neurosci. **38**, 8707–8722 (2018), 10.1523/JNEUROSCI.0734-18.2018. 30201775 PMC6596226

[27] E. Ivanova , AAV-BR1 targets endothelial cells in the retina to reveal their morphological diversity and to deliver Cx43. J. Comp. Neurol. **530**, 1302–1317 (2022), 10.1002/cne.25277. 34811744 PMC8969189

[28] A. A. Berthiaume , Dynamic remodeling of pericytes in vivo maintains capillary coverage in the adult mouse brain. Cell Rep. **22**, 8–16 (2018), 10.1016/j.celrep.2017.12.016. 29298435 PMC5782812

[29] A. A. Berthiaume , Pericyte remodeling is deficient in the aged brain and contributes to impaired capillary flow and structure. Nat. Commun. **13**, 5912 (2022), 10.1038/s41467-022-33464-w. 36207315 PMC9547063

[30] K. Bisht , Capillary-associated microglia regulate vascular structure and function through PANX1-P2RY12 coupling in mice. Nat. Commun. **12**, 5289 (2021), 10.1038/s41467-021-25590-8. 34489419 PMC8421455

[31] T. R. F. Green, S. M. Murphy, R. K. Rowe, Comparisons of quantitative approaches for assessing microglial morphology reveal inconsistencies, ecological fallacy, and a need for standardization. Sci. Rep. **12**, 18196 (2022), 10.1038/s41598-022-23091-2. 36307475 PMC9616881

[32] S. J. van Veluw , Vasomotion as a driving force for paravascular clearance in the awake mouse brain. Neuron **105**, 549–561.e5 (2020), 10.1016/j.neuron.2019.10.033. 31810839 PMC7028316

[33] C. Cai , Stimulation-induced increases in cerebral blood flow and local capillary vasoconstriction depend on conducted vascular responses. Proc. Natl. Acad. Sci. U.S.A. **115**, E5796–E5804 (2018), 10.1073/pnas.1707702115. 29866853 PMC6016812

[34] E. F. Hauck, S. Apostel, J. F. Hoffmann, A. Heimann, O. Kempski, Capillary flow and diameter changes during reperfusion after global cerebral ischemia studied by intravital video microscopy. J. Cereb. Blood Flow Metab. **24**, 383–391 (2004), 10.1097/00004647-200404000-00003. 15087707

[35] L. Khennouf , Active role of capillary pericytes during stimulation-induced activity and spreading depolarization. Brain **141**, 2032–2046 (2018), 10.1093/brain/awy143. 30053174 PMC6022680

[36] Ş Erdener, G. Küreli, T. Dalkara, Contractile apparatus in CNS capillary pericytes. Neurophotonics **9**, 021904 (2022), 10.1117/1.NPh.9.2.021904. 35106320 PMC8785978

[37] H. K. Shin, S. Salomone, C. Ayata, Targeting cerebrovascular Rho-kinase in stroke. Expert Opin. Ther. Targets **12**, 1547–1564 (2008), 10.1517/14728220802539244. 19007322

[38] J. Zhao , Effect of fasudil hydrochloride, a protein kinase inhibitor, on cerebral vasospasm and delayed cerebral ischemic symptoms after aneurysmal subarachnoid hemorrhage. Neurol. Med. Chir. (Tokyo) **46**, 421–428 (2006), 10.2176/nmc.46.421. 16998274

[39] M. Barton, M. Yanagisawa, Endothelin: 30 years from discovery to therapy. Hypertension **74**, 1232–1265 (2019), 10.1161/HYPERTENSIONAHA.119.12105. 31679425

[40] A. Kowalczyk, P. Kleniewska, M. Kolodziejczyk, B. Skibska, A. Goraca, The role of endothelin-1 and endothelin receptor antagonists in inflammatory response and sepsis. Arch. Immunol. Ther. Exp. (Warsz) **63**, 41–52 (2015), 10.1007/s00005-014-0310-1. 25288367 PMC4289534

[41] Y. Miyoshi , Endothelin blocks ATP-sensitive K+ channels and depolarizes smooth muscle cells of porcine coronary artery. Circ. Res. **70**, 612–616 (1992), 10.1161/01.res.70.3.612. 1537097

[42] W. S. Park, E. A. Ko, J. Han, N. Kim, Y. E. Earm, Endothelin-1 acts via protein kinase C to block KATP channels in rabbit coronary and pulmonary arterial smooth muscle cells. J. Cardiovasc. Pharmacol. **45**, 99–108 (2005), 10.1097/01.fjc.0000150442.49051.f7. 15654257

[43] M. S. Thomsen, L. J. Routhe, T. Moos, The vascular basement membrane in the healthy and pathological brain. J. Cereb. Blood Flow Metab. **37**, 3300–3317 (2017), 10.1177/0271678X17722436. 28753105 PMC5624399

[44] P. C. Nahirney, P. Reeson, C. E. Brown, Ultrastructural analysis of blood-brain barrier breakdown in the peri-infarct zone in young adult and aged mice. J. Cereb. Blood Flow Metab. **36**, 413–425 (2016), 10.1177/0271678X15608396. 26661190 PMC4759675

[45] T. A. Longden , Local IP3 receptor-mediated Ca2+ signals compound to direct blood flow in brain capillaries. Sci. Adv. **7**, eabh0101 (2021), 10.1126/sciadv.abh0101. 34290098 PMC8294755

[46] J. Steinman, H. S. Sun, Z. P. Feng, Microvascular alterations in Alzheimer’s disease. Front. Cell Neurosci. **14**, 618986 (2020), 10.3389/fncel.2020.618986. 33536876 PMC7849053

[47] C. Cai , Impaired dynamics of precapillary sphincters and pericytes at first-order capillaries predict reduced neurovascular function in the aging mouse brain. Nat. Aging. **3**, 173–184 (2023), 10.1038/s43587-022-00354-1. 37118115 PMC11081516

[48] P. Shin , Aerobic exercise reverses aging-induced depth-dependent decline in cerebral microcirculation. eLife **12**, e86329 (2023), 10.7554/eLife.86329. 37402178 PMC10319437

[49] A. S. Cuttler , Characterization of Pdgfrb-Cre transgenic mice reveals reduction of ROSA26 reporter activity in remodeling arteries. Genesis **49**, 673–680 (2011), 10.1002/dvg.20769. 21557454 PMC3244048

[50] L. Madisen , A robust and high-throughput Cre reporting and characterization system for the whole mouse brain. Nat. Neurosci. **13**, 133–140 (2010), 10.1038/nn.2467. 20023653 PMC2840225

[51] L. Tian , Imaging neural activity in worms, flies and mice with improved GCaMP calcium indicators. Nat. Methods **6**, 875–881 (2009), 10.1038/nmeth.1398. 19898485 PMC2858873

[52] S. Jung , Analysis of fractalkine receptor CX(3)CR1 function by targeted deletion and green fluorescent protein reporter gene insertion. Mol. Cell Biol. **20**, 4106–4114 (2000), 10.1128/MCB.20.11.4106-4114.2000. 10805752 PMC85780

[53] B. Li , Two-photon microscopic imaging of capillary red blood cell flux in mouse brain reveals vulnerability of cerebral white matter to hypoperfusion. J. Cereb. Blood Flow Metab. **40**, 501–512 (2020), 10.1177/0271678X19831016. 30829101 PMC7026840

[54] K. P. McDowell, A. A. Berthiaume, T. Tieu, D. A. Hartmann, A. Y. Shih, VasoMetrics: Unbiased spatiotemporal analysis of microvascular diameter in multi-photon imaging applications. Quant. Imaging Med. Surg. **11**, 969–982 (2021), 10.21037/qims-20-920. 33654670 PMC7829163

[55] T. N. Kim , Line-scanning particle image velocimetry: An optical approach for quantifying a wide range of blood flow speeds in live animals. PLoS One **7**, e38590 (2012), 10.1371/journal.pone.0038590. 22761686 PMC3383695

[56] S. K. Bonney , Data from “Capillary regression leads to sustained local hypoperfusion by inducing constriction of upstream transitional vessels.” OSF. https://osf.io/xzt6r/. Deposited 20 August 2024. 10.1073/pnas.2321021121 PMC11406265 39236241

