# Supplementary material for: Capillary regression leads to sustained local hypoperfusion by inducing constriction of upstream transitional vessels
Source: Proc Natl Acad Sci U S A. 2024 Sep 5;121(37):e2321021121. doi: 10.1073/pnas.2321021121 (PMC11406265; doi:10.1073/pnas.2321021121)
Supplement: Supplementary file 1 — Appendix 01 (PDF) [file pnas.2321021121.sapp.pdf]

**SUPPLEMENTAL INFORMATION FOR:**

# Capillary regression leads to sustained local hypoperfusion by inducing constriction of upstream transitional vessels

Stephanie K. Bonney, Cara D. Nielson, Maria J. Sosa, Orla Bonnar, and Andy Y. Shih

## SUPPLEMENTAL MATERIALS & METHODS

*Extended details for capillary injury using two-photon irradiation.* Initial experiments were performed to optimize the amount of laser power, irradiation time, and rounds of line-scanning required for rupturing a capillary, depending on cortical depth. This allowed us to create a scatter plot to predict how much power was needed to rupture a capillary for following experiments (**Supp. Fig. 1F**). Laser ablations were initiated with the lowest power predicted for the target vessel depending on cortical depth, and gradually ramped as needed. In between each 20s line-scan we monitored the blood flow and diameter of the target vessel to determine if the vessel was weakening, or if laser power needed to be increased by an increment of ~50mW for the subsequent 20s line-scans. Once blood flow was diminishing or the vessel was constricting, the remainder of the 20s line-scan rounds were maintained at that respective power. Using this method, vessels generally ruptured within 80s of focusing the line-scans at the minimum amount of power needed to weaken the vessel.

*Endothelial cell labeling.* To label the brain endothelium, we retro-orbitally administered 20 μL of the endothelial-specific adeno-associated viral vector, AAV-BR1-GFP, combined with 30 μL phosphate buffered saline, into a PdgfrβCre-tdTomato mouse with a cranial window[1]. Following the incubation period of two weeks to allow for widespread expression of GFP in the endothelium, we performed capillary injuries. The injured capillaries were re-imaged at 14 dpi to examine endothelial changes at the injury site.

*Analysis of pericyte remodeling, vessel coverage, and pericyte-associated diameter changes.* To analyze pericyte remodeling following capillary injury-induced pericyte death, remodeling processes were first identified by determining which processes of neighboring pericytes had lost pericyte contact at their terminal tips. Then, using the simple neurite tracer (SNT) plugin in FIJI, process length was measured from soma to process terminus at each imaging time point[2]. Process extension was calculated by subtracting process length at baseline from process length at each imaging time point. Vascular length uncovered by pericytes at each time point was also measured using SNT in FIJI and percent vessel coverage was based on uncovered vessel length 1 dpi. Vessel diameter was determined in pre-injury images (prior to pericyte death), the uncovered state (3 dpi) and the recovered state (14 dpi) using the VasoMetrics FIJI plugin[3].

*Analysis of microglia morphology*. In images from PdgfrβCre-tdTomato; Cx3cr1-GFP mice, $GFP^+$ cells with thin, ramified cellular processes[4], presumably microglia, surrounding the ACT zone were analyzed pre-, 3-, and 14-days post injury. The types of microglia-vessel interactions in the ACT

zone, i.e., processes or soma contact, were noted at each imaging time point[5]. The processes extending from the soma of each ACT-associated microglia were traced in 3D z-stacks using the SNT plugin in FIJI in the green channel, and 3-4 microglia per ACT zone per time point was examined. Each microglia trace was then max projected into a 2D image and Sholl analysis was performed with 5µm intervals set for the concentric circles up to 100µm away from the microglia soma. The number of intersections at each 5µm interval, the total number of processes, and the total process length were extracted for each microglia at each time point. Identical analyses were performed on microglia surrounding the injury site 1-day post injury to demonstrate how the elaboration of microglia processes decrease during injury and inflammation.

*Analysis of mural cell calcium and vasomotor frequency and power*. The mean frequency and power of the calcium and diameter time-series were calculated in MATLAB using the meanfreq() and bandpower() functions, respectively, for frequencies in the 0.025–0.2 Hz range, the frequency band known to represent vasomotor activity[6]. Time-series that were too short to resolve frequencies in this band (<80s) were excluded from analysis. Data shown in Supplemental Figures 8A and 9B are smoothed data with a running average window of 5 data points, corresponding to 2.048 s to better show the trend of $Ca^{2+}$ signatures and vasomotion.

**REFERENCES**

1. Ivanova E, Corona C, Eleftheriou CG, Stout RF, Körbelin J, Sagdullaev BT. AAV-BR1 targets endothelial cells in the retina to reveal their morphological diversity and to deliver Cx43. *J Comp Neurol*. Jun 2022;530(8):1302-1317. doi:10.1002/cne.25277
2. Berthiaume AA, Grant RI, McDowell KP, et al. Dynamic Remodeling of Pericytes In Vivo Maintains Capillary Coverage in the Adult Mouse Brain. *Cell Rep*. 01 2018;22(1):8-16. doi:10.1016/j.celrep.2017.12.016
3. McDowell KP, Berthiaume AA, Tieu T, Hartmann DA, Shih AY. VasoMetrics: unbiased spatiotemporal analysis of microvascular diameter in multi-photon imaging applications. *Quant Imaging Med Surg*. Mar 2021;11(3):969-982. doi:10.21037/qims-20-920
4. Jurga AM, Paleczna M, Kuter KZ. Overview of General and Discriminating Markers of Differential Microglia Phenotypes. *Front Cell Neurosci*. 2020;14:198. doi:10.3389/fncel.2020.00198
5. Bisht K, Okojie KA, Sharma K, et al. Capillary-associated microglia regulate vascular structure and function through PANX1-P2RY12 coupling in mice. *Nat Commun*. Sep 06 2021;12(1):5289. doi:10.1038/s41467-021-25590-8
6. van Veluw SJ, Hou SS, Calvo-Rodriguez M, et al. Vasomotion as a Driving Force for Paravascular Clearance in the Awake Mouse Brain. *Neuron*. 02 2020;105(3):549-561.e5. doi:10.1016/j.neuron.2019.10.033

# SUPPLEMENTAL FIGURES

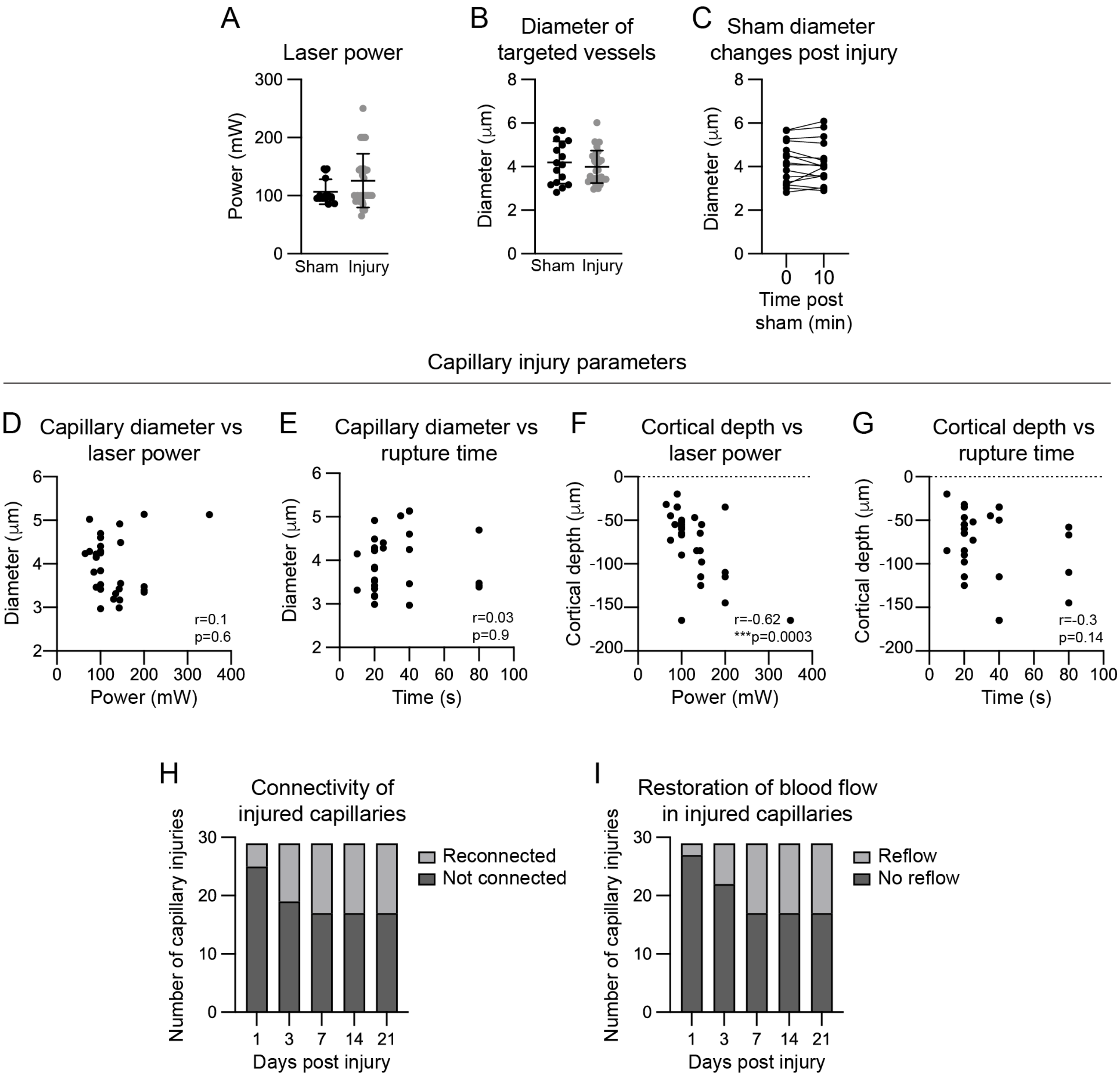


**Supplemental Figure 1. Parameters for laser-induced capillary injury.**

**(A, B)** Graphs of (A) applied laser power and (B) vessel diameter of sham (black) and capillary (gray) injuries. Appropriate parametric and non-parametric tests were used depending on distribution of data. No significant differences were detected between groups. 29 capillary injury experiments conducted over 15 mice. Sham injuries = 17 conducted over 13 mice.

**(C)** Graph of vessel diameter changes for capillaries adjacent to sham injury pre and 10-minutes post injury. No significant difference detected by paired t-test.
**(D, E)** Scatter plots of vessel diameter versus (D) laser power and (E) rupture time to induce capillary injuries. Spearman's rank correlations were performed, respective r and p values are reported on graphs.
**(F, G)** Scatter plots of cortical depth versus (F) laser power and (G) rupture time to induce capillary injuries. Pearson correlation test demonstrates an increase in laser power is needed to rupture capillaries deeper into the cortex.
**(H)** Graph showing the number of capillary injuries (total of 29) where vessels were reconnected (light gray) or not connected (dark gray) for up to 21 days post capillary injury.
(**I**) Graph showing when blood flow was reestablished (light gray) or not (dark gray) for up to 21 days post capillary injury.

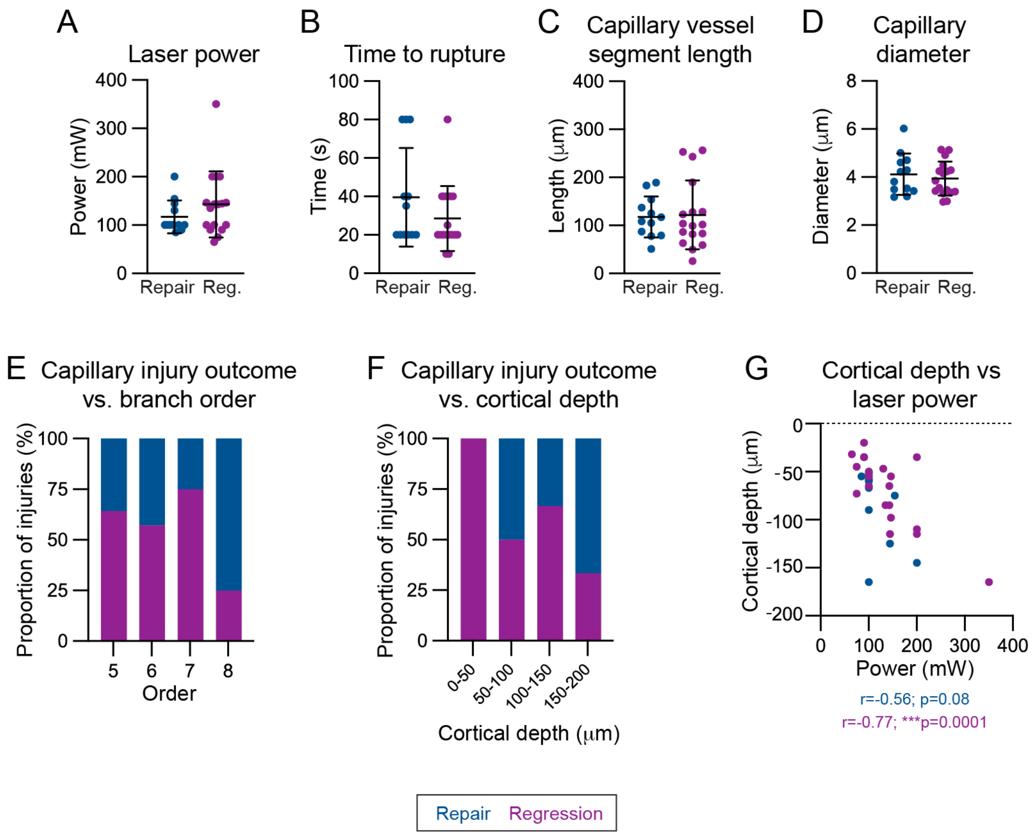


**Supplemental Figure 2. Comparison of parameters for capillary injury between regression and repair events.**

**(A-D)** Graphs of (A) applied laser power, (B) rupture time, (C) vessel segment length, and (D) vessel diameter for capillaries that repaired (blue; n=12) or regressed (Reg.; purple; n=17). Appropriate parametric and non-parametric tests were used depending on distribution of data and no significant differences were detected.

**(E, F)** Graphs showing the proportion of capillary injuries that resulted in vessel repair or regression in relation to (E) vessel branch order from the penetrating arteriole and (F) cortical depth. Repair: 5$^{th}$ order: n=5, 6$^{th}$ order: n=3, 7$^{th}$ order: n=1, 8$^{th}$ order: n=3; Regression: 5$^{th}$ order: n=9, 6$^{th}$ order: n=4, 7$^{th}$ order: n=3, 8$^{th}$ order: n=1. Repair: 0-50µm: n=0, 50-100µm: n=8, 100-150µm: n=2, 150-200µm: n=2. Regression: 0-50µm: n=4, 50-100µm: n=8, 100-150µm: n=4, 150-200µm: n=1.

(**G**) Scatter plots of laser power versus cortical depth for regression and repair events demonstrating deeper capillaries required higher laser powers to rupture. Pearson correlations were performed, respective r and p values for repair and regression groups are reported with graph.

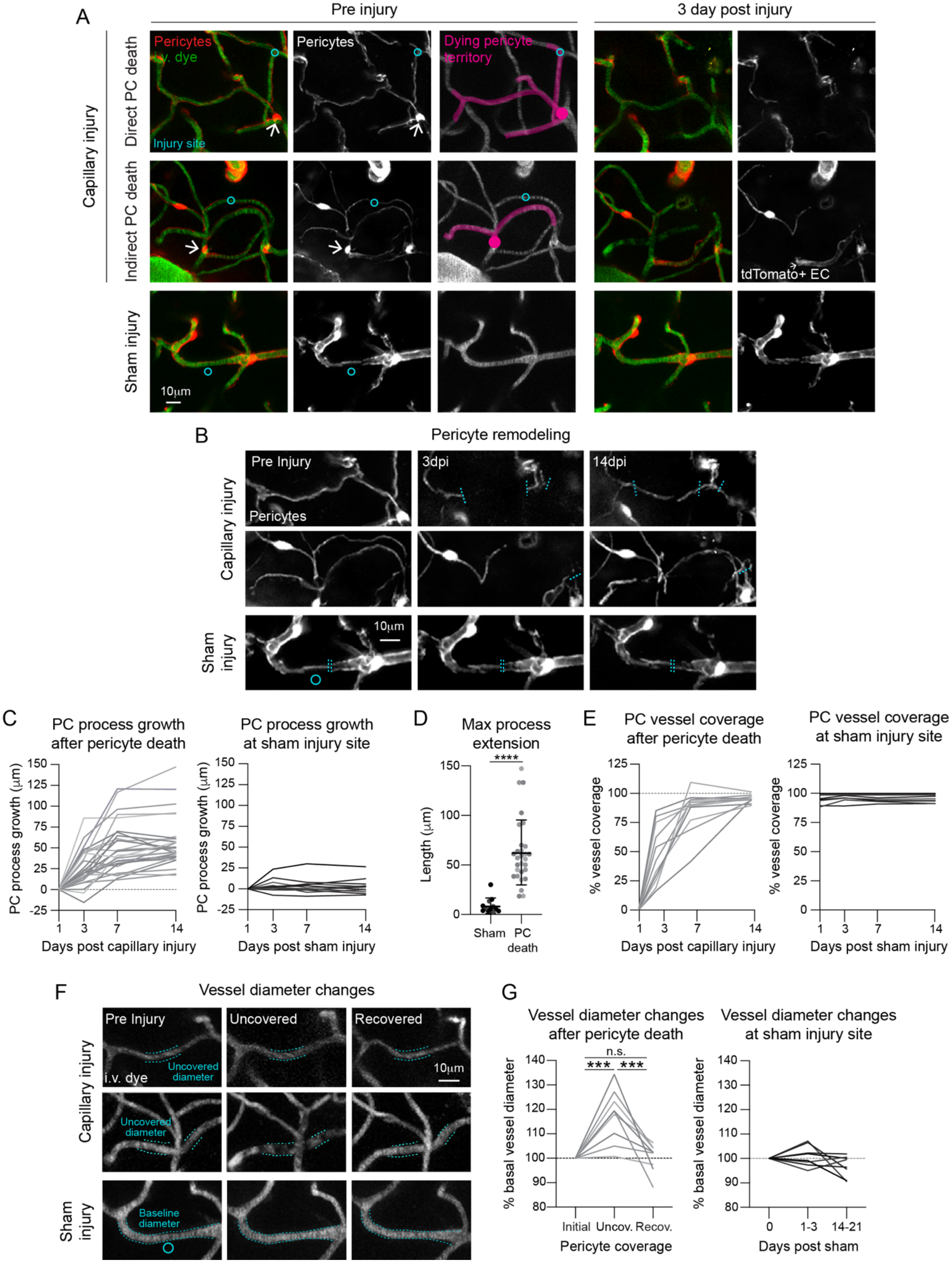

A
Pre injury
3 day post injury
Capillary injury
Direct PC death
Indirect PC death
Sham injury
Pericytes
i.v. dye
Injury site
Pericytes
Dying pericyte territory
tdTomato+ EC
10µm
B
Pericyte remodeling
Pre Injury
3dpi
14dpi
Pericytes
Capillary injury
Sham injury
10µm
C
PC process growth after pericyte death
PC process growth at sham injury site
PC process growth (µm)
Days post capillary injury
Days post sham injury
D
Max process extension
Length (µm)
****
Sham
PC death
E
PC vessel coverage after pericyte death
PC vessel coverage at sham injury site
% vessel coverage
Days post capillary injury
Days post sham injury
F
Vessel diameter changes
Pre Injury
Uncovered
Recovered
i.v. dye
Uncovered diameter
10µm
Capillary injury
Sham injury
Baseline diameter
G
Vessel diameter changes after pericyte death
Vessel diameter changes at sham injury site
% basal vessel diameter
n.s.
***
***
Initial
Uncov.
Recov.
Pericyte coverage
Days post sham

**Supplemental Figure 3. Pericyte death may be induced by capillary injury, but remodeling of neighboring pericytes ensures coverage.**

**(A)** Representative *in vivo* images of capillary injuries (cyan circle) in a PdgfrβCre-tdTomato mouse pre and 3 days post injury that resulted in pericyte death. Direct pericyte death occurred when line-scan path injured the pericyte process (upper panel). Indirect pericyte death occurred when a nearby, uninjured pericyte died following capillary injury (middle panel). Territories of dying pericytes demonstrated in pink. Sham injuries did not induce pericyte death (lower panel). Pericytes depicted in red and grayscale with i.v. dye (70kDa FITC-Dextran) in green. Arrows indicate pericytes that died by 3 days post injury. Note: tdTomato+ endothelial cells are occasionally apparent in the PdgfrβCre-tdTomato mouse line.

**(B)** Representative *in vivo* images of pericyte remodeling following capillary injuries (upper and middle panels) and pericyte process movement following sham injuries (lower panels). Cyan dashed lines indicate the end of neighboring pericyte processes 3 days post capillary injury.

**(C)** Graphs of neighboring pericyte (PC) process growth over the course of 14 days following pericyte death (gray; left) and sham injuries (black; right). Pericyte death occurred in 7/29 of capillary injuries (in 4 mice). Sham injuries: n=8 pericyte processes, 5 mice.

**(D)** Graph of maximum process extension of pericyte processes following pericyte death and sham injuries. Mann-whitney U test detected significant differences between pericyte death and sham injuries ****$p<0.0001$.

**(E)** Graphs of pericyte (PC) vessel coverage over the course of 14 days following pericyte death (left) and sham injuries (right).

**(F)** Representative *in vivo* images of vessel diameter changes pre-injury, post pericyte death (uncovered), and when vessels were recovered by pericyte processes following capillary (upper 2 panels) and sham (lower panel) injuries. Dashed lines outline uncovered vessel diameter size in uncovered state for capillary injuries and baseline diameter for sham injuries. Images in A, B, and F are representatives from the same experiments for both pericyte death examples and sham experiments.

**(G)** Graphs of vessel diameter changes during pre-injury, uncovered, and recovered states for capillary injuries that resulted in pericyte death (left). Sham injuries (right) show diameter changes at 1-3 and 14-21-days post sham. ANOVA followed by Tukey's multiple comparison tests were performed: pericyte death: initial vs. uncovered ***$p=0.0002$, uncovered vs. recovered ***$p=0.0003$.

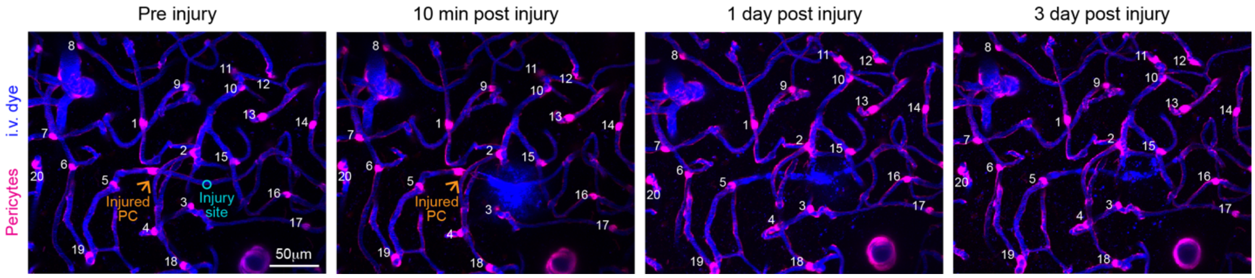


**Supplemental Figure 4. Injured pericytes do not migrate following capillary injury.**
**(A)** Representative *in vivo* images of an injured and dying pericyte (orange arrow) following capillary injury (cyan circle) in a PdgfrβCre-tdTomato mouse pre, 10 mins-, 1 day- and 3 days-post injury. Surrounding pericytes are numbered 1-20 and demonstrate the stability of the neighboring pericytes following capillary injury while the injured pericyte is dying. By 1 day post injury we observe no pericytes within the surrounding parenchyma detached from the vessels and the injured pericyte now gone. We conclude that this pericyte most likely died following capillary injury. Pericytes depicted in red with i.v. dye (2MDa Alexa-680) in blue.

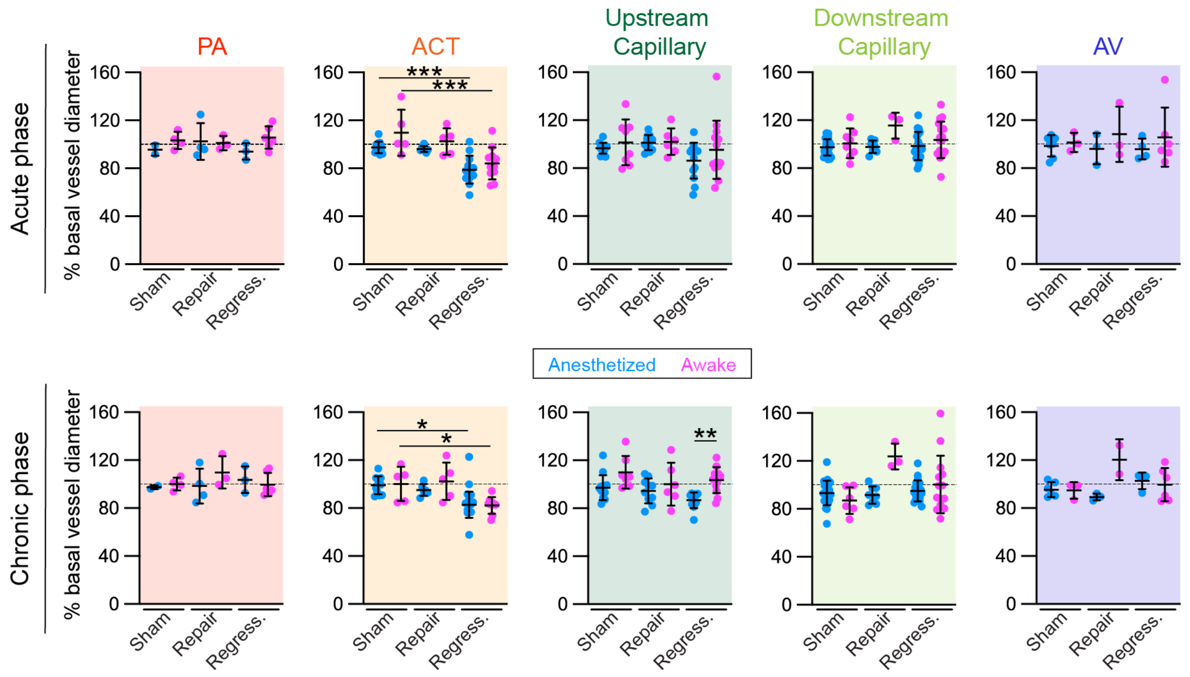


**Supplemental Figure 5. Comparison of vessel diameter changes along the vascular zones following sham, repair, and regression events in anesthetized and awake mice.**
Graphs of percent change in the diameter of vessel segments throughout the microvascular zones encompassing penetrating arterioles (PA), arteriole-capillary transition zones (ACT), capillaries, and ascending venules (AV) of sham and repair events in animals that underwent anesthetized (blue) or awake (pink) imaging. Change from pre-injury is shown during the acute (3 or 7 days) and chronic (14 or 21 days) phase following capillary or sham injury. ANOVA followed by Tukey's or Dunn's multiple comparison tests were performed depending on distribution of data. ACT zone: Acute: anesthetized sham vs. regression ***p=0.0005; awake sham vs. regression ***p=0.0004. Chronic: anesthetized sham vs. regression *p=0.04; awake sham vs. regression *p=0.04. Upstream capillary: Chronic: anesthetized regression vs. awake regression **p=0.004. Each datapoint is the diameter from a single vessel segment. Anesthetized: sham = 6 experiments in 4 mice; repair = 4 experiments in 4 mice; regression = 9 experiments in 6 mice; Awake: sham = 4 experiments in 4 mice; repair = 3 experiments in 3 mice; regression = 6 experiments in 4 mice.

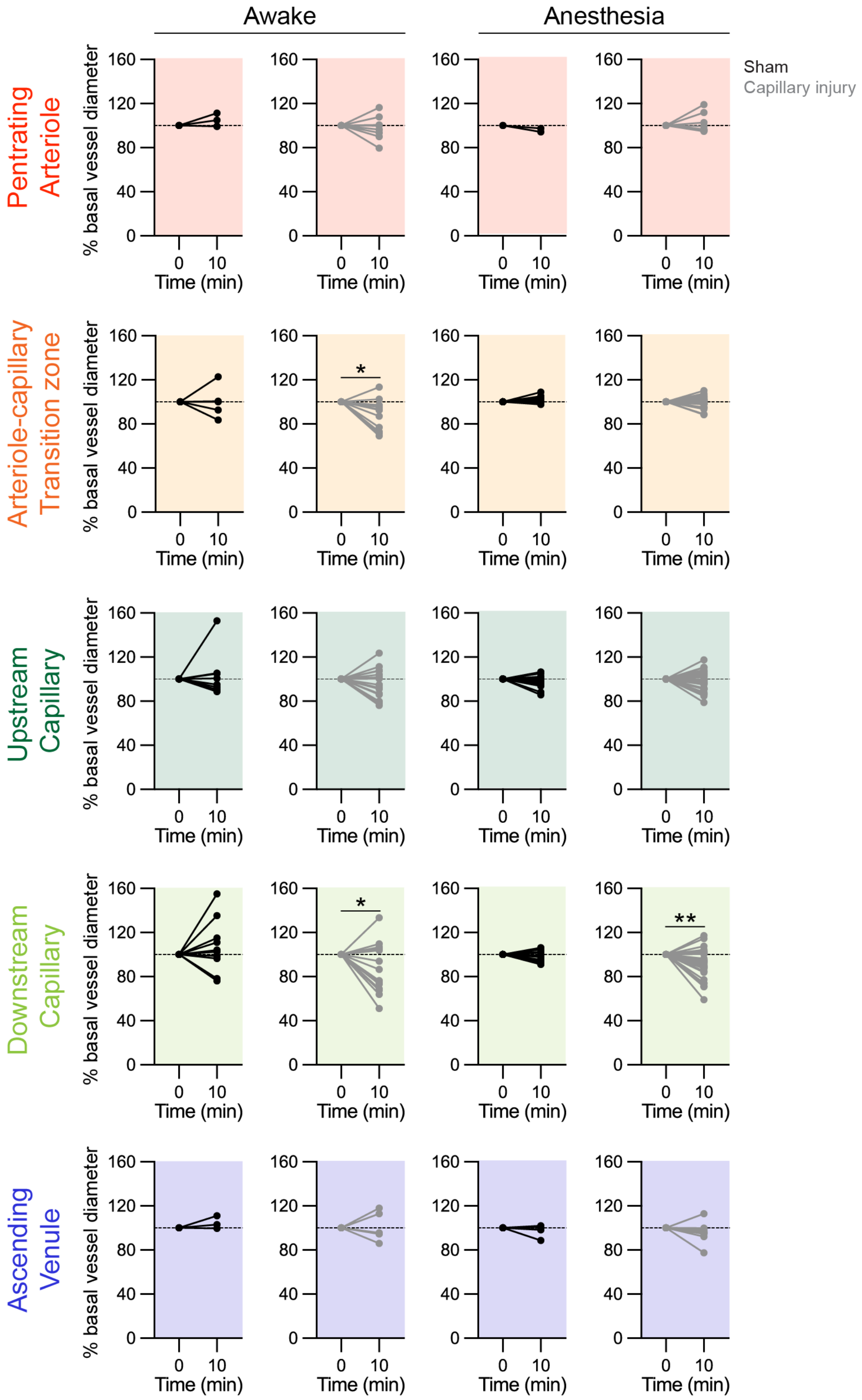


**Supplemental Figure 6. Arteriole-capillary transition vessels constrict as early as 10 minutes post-optical capillary injury in awake animals.**

Graphs of percent change in the diameter of vessel segments 10 minutes post capillary injury or sham throughout the microvascular zones, encompassing penetrating arterioles (PA), arteriole-capillary transition zones (ACT), capillaries, and ascending venules (AV) of sham and repair events in animals that underwent anesthetized (blue) or awake (pink) imaging. Change from pre-injury is shown during the acute (3 or 7 days) and chronic (14 or 21 days) phase following capillary or sham injury. Paired t-tests were performed. ACT zone: Awake sham vs. capillary injury *p=0.015. Downstream capillary: Awake sham vs. capillary injury *p=0.03; Anesthesia sham vs. capillary injury **p=0.001. Each datapoint is the diameter from a single vessel segment. Anesthetized: sham = 6 experiments in 4 mice; capillary injuries = 14 experiments in 7 mice. Awake: sham = 4 experiments in 4 mice; capillary injury = 8 experiments in 5 mice.

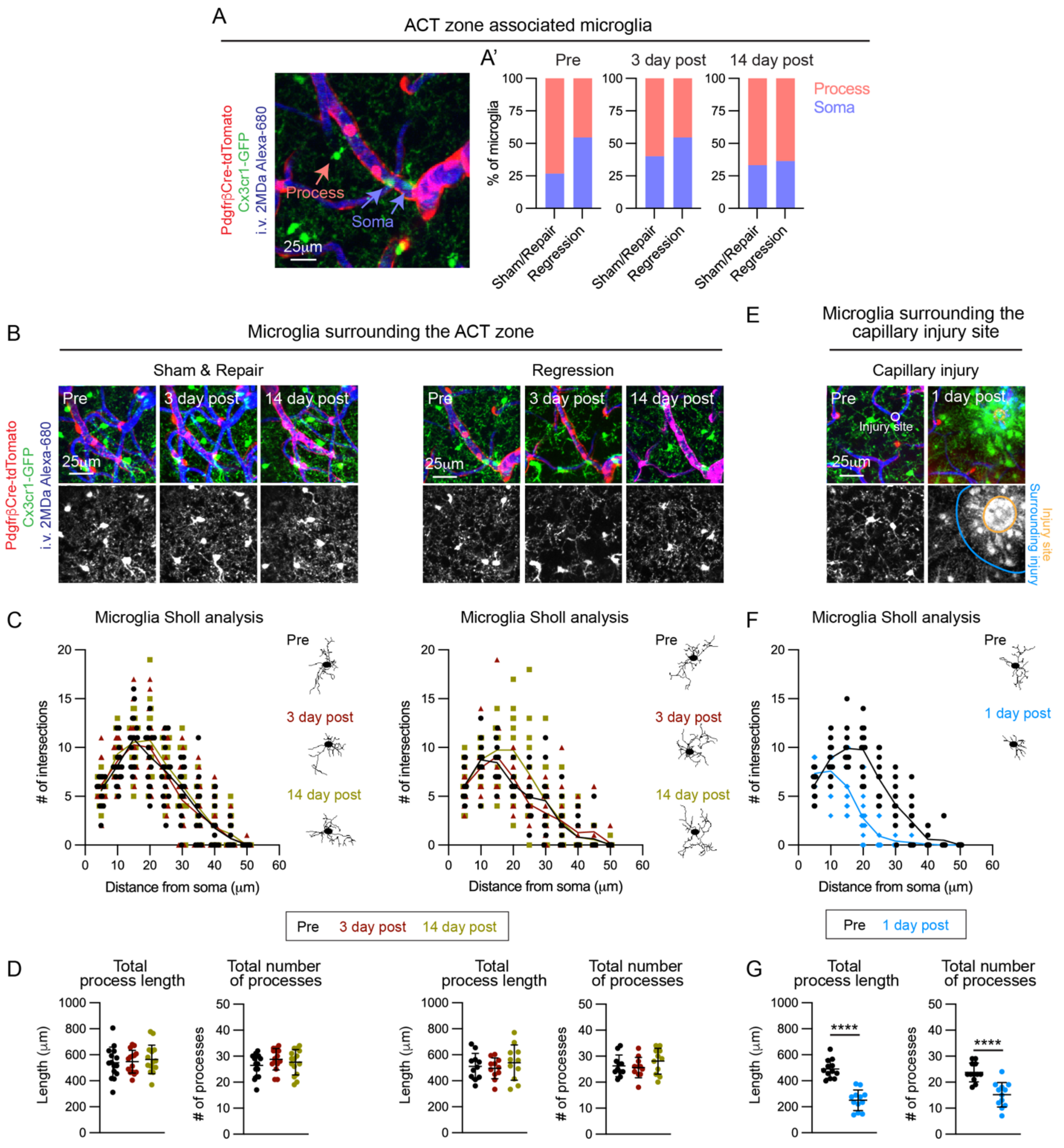


**Supplemental Figure 7. Microglia surrounding the ACT zone are not activated during capillary regression.**

**(A)** Representative *in vivo* images of microglia associated with the ACT zone either via their soma (light purple) or processes (light orange) in a PdgfrβCre-tdTomato; Cx3Cr1-GFP mouse prior to injury. (**A'**) Graphs demonstrating that microglia associations via their soma and processes does not change 3- and

14-day post sham/repair and regression events. Pericytes depicted in red, microglia in green, with i.v. dye (2MDa Alexa-680) in blue.
**(B)** Representative *in vivo* images of microglia associated with the ACT zone in Sham/Repair and Regression events demonstrating microglia morphology does not drastically change following downstream capillaries regression.
(**C**) Scatter plots of Sholl analysis on the number of microglia process intersections crossing concentric circles increasing by 5μm radially from the soma of microglia surrounding ACT zones in sham/repair (left) and regression (right). This demonstrates the elaboration of microglia processes does not change following sham, repair, and regression events 3- and 14-days post injury. Pre-injury shown in black, 3-day in maroon, 14-day in gold. Third-order polynomial best fit lines are shown with the number of intersections at each 5μm interval to demonstrate the trend in microglia process elaboration surrounding the ACT zone.
(**D**) Graphs of the total process length and total process number on microglia surrounding the ACT zone pre, 3-, and 14-day post sham/repair (left) and regression (right) injury events. ANOVA analysis was performed and no significant differences were detected.
**(E)** Representative *in vivo* images of microglia surrounding a capillary injury site pre and 1 day post injury. Injury site indicated in yellow with clear individual microglia surrounding the injury site (blue outline).
(**F**) Scatter plots of Sholl analysis on the number of microglia process intersections crossing concentric circles increasing by 5μm radially from the soma of microglia surrounding the capillary injury site 1 day post injury (blue). This demonstrates the elaboration of microglia processes decreases in microglia surrounding injury site suggesting an activated morphology.
(**G**) Graphs of the total process length and total process number on microglia surrounding the ACT zone pre and 1 day post capillary injury. Appropriate parametric and non-parametric t tests were performed depending on the distribution of the data. Total process length: Mann Whitney test: ****$p<0.0001$. Total branch length: Unpaired t test: ****$p<0.0001$.

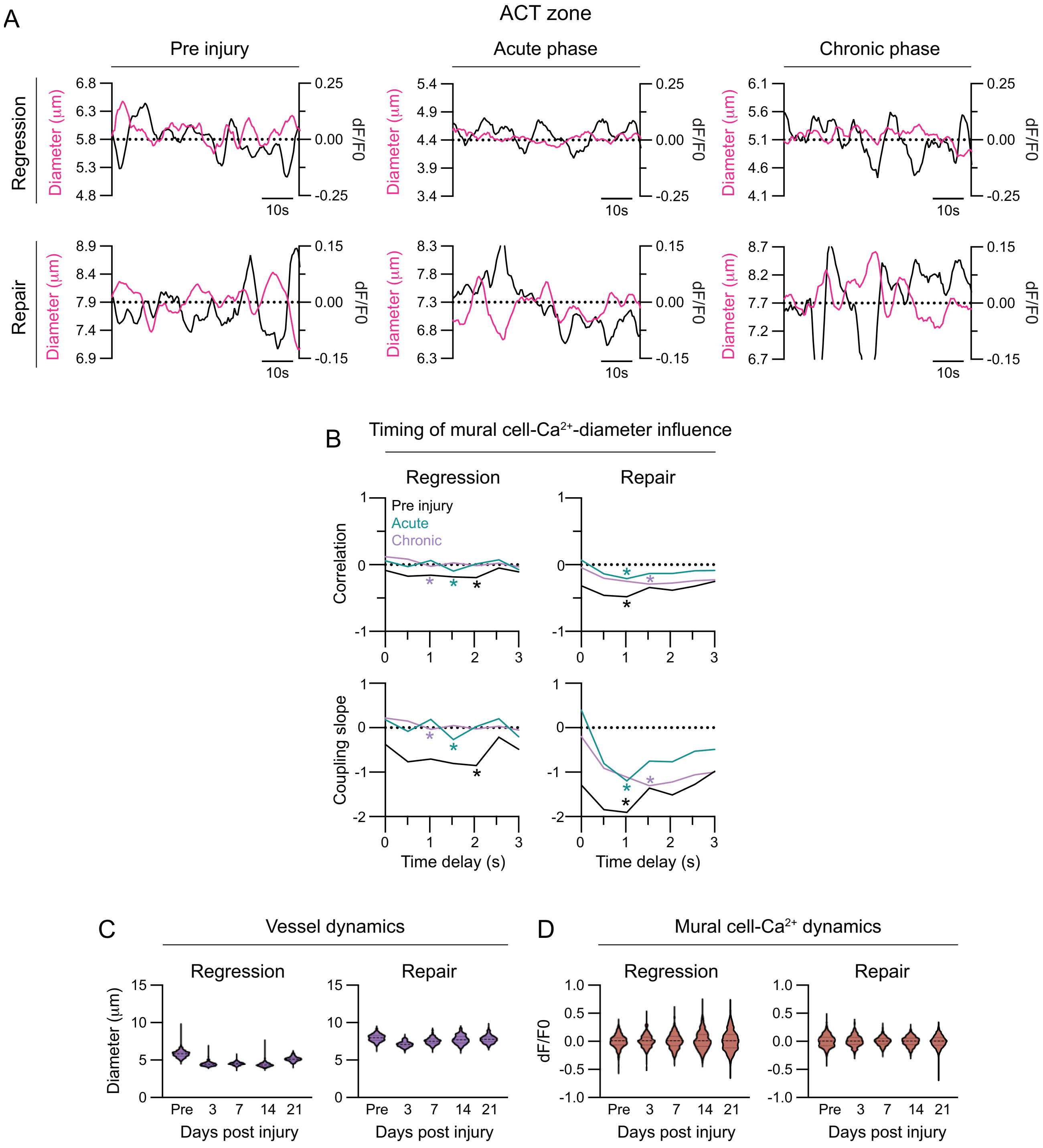


**Supplemental Figure 8. Mural cell calcium signaling and vessel diameter dynamics in arteriole-capillary transition zone following capillary injury.**

**(A)** Graphs showing case examples of change in arteriole-capillary transition (ACT) zone vessel diameter and mural cell calcium signaling (dF/F0) over 1 minute prior to injury and during the acute (3 or 7 days) and chronic (14 or 21 days) in a capillary regression and repair event.

**(B)** Graphs showing case examples of cross correlation analysis and respective coupling slopes within 3 seconds of ACT zone vessel diameter and mural cell-$Ca^{2+}$ signaling changes for a capillary regression and repair event. Strongest influence of mural cell-GCaMP6f signaling on ACT vasodynamics are indicated with asterisks and based on the strongest correlation for experiments pre-injury (black) and in the acute (green) and chronic (purple) phase.
**(C, D)** Graphs showing the distribution of (C) vessel diameter and (D) mural cell calcium signaling (dF/F0) in the ACT zone over at least 2 minutes prior to injury and 3-, 7-, 14-, 21-days post injury in a regression and repair event.

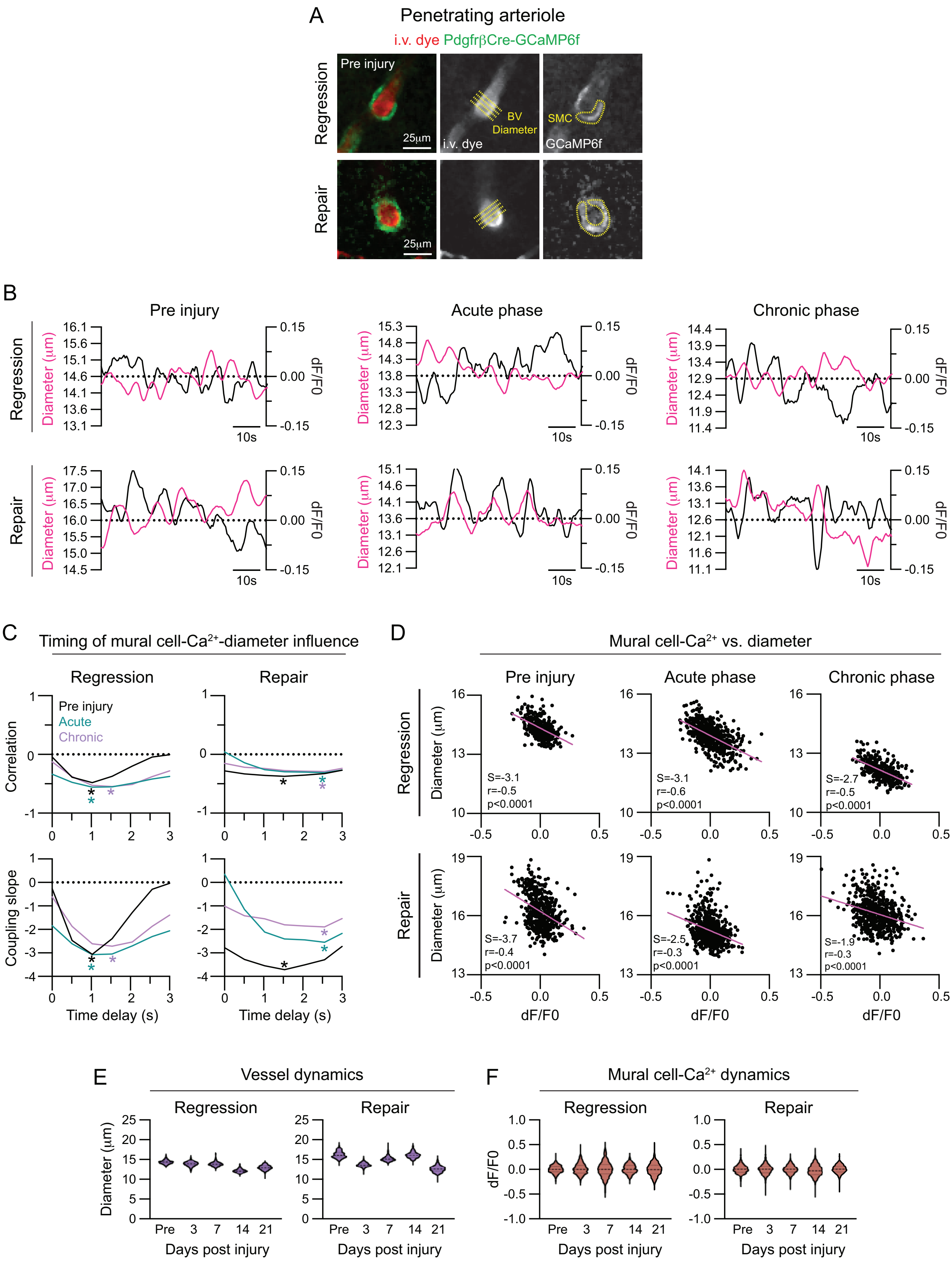
A
Penetrating arteriole
i.v. dye PdgfrβCre-GCaMP6f
Regression
Repair
Pre injury
25μm
BV Diameter
i.v. dye
SMC
GCaMP6f
B
Pre injury
Acute phase
Chronic phase
Regression
Repair
Diameter (μm)
dF/F0
10s
C
Timing of mural cell-Ca2+-diameter influence
Regression
Repair
Pre injury
Acute
Chronic
Correlation
Coupling slope
Time delay (s)
D
Mural cell-Ca2+ vs. diameter
Pre injury
Acute phase
Chronic phase
Regression
Repair
Diameter (μm)
dF/F0
S=-3.1
r=-0.5
p<0.0001
S=-3.1
r=-0.6
p<0.0001
S=-2.7
r=-0.5
p<0.0001
S=-3.7
r=-0.4
p<0.0001
S=-2.5
r=-0.3
p<0.0001
S=-1.9
r=-0.3
p<0.0001
E
Vessel dynamics
Regression
Repair
Diameter (μm)
Pre 3 7 14 21
Days post injury
F
Mural cell-Ca2+ dynamics
Regression
Repair
dF/F0
Pre 3 7 14 21
Days post injury

**Supplemental Figure 9. Mural cell calcium signaling and vessel diameter dynamics are not altered along penetrating arterioles following capillary injury.**

**(A)** Representative *in vivo* t-series image of penetrating arteriole (PA) from a regression and repair event in an awake PdgfrβCre-GCamP6f mouse. Smooth muscle cells (SMCs) are shown in green and i.v. dye (70kDa Texas Red-Dextran) labeling vessels depicted in red. In respective grayscale images, an example of vessel diameter (yellow crosslines) and GCaMP6 fluorescent intensity (yellow outline) analysis is shown.

**(B)** Graphs showing case examples of change in PA vessel diameter and mural cell calcium signaling (dF/F0) over 1 minute prior to injury and during the acute (3 or 7 days) and chronic (14 or 21 days) phases in a capillary regression and repair event.

**(C)** Graphs showing case examples of cross correlation analysis and respective coupling slopes within 3 seconds of PA vessel diameter and mural cell calcium signaling changes in a capillary regression and repair event. Strongest influence of mural cell calcium signaling on PA vasodynamics are indicated with asterisks and based on the strongest correlation for experiments pre-injury (black) and in the acute (green) and chronic (purple) phase.

**(D)** Scatter plots of change in mural cell calcium signal (dF/F0) versus PA diameter over at least 2 minutes (data point collected every 0.512s or 1.951Hz) pre-injury, acute (3- or 7-days post injury) and chronic (14- or 21-days post injury) phase. Plots are shown following cross-correlation analysis (Supplemental Fig. 9C) with strongest correlation. Regression correlation time: Pre t=1.024s, Acute t=1.024s, Chronic t=1.536s; Repair correlation time: Pre t=1.536s, Acute t=2.56s, Chronic t=2.56s. Pearson correlation tests were performed, respective r and p values are reported on graphs along with the coupling slope (S).

**(E, F)** Graphs showing distribution of (E) vessel diameter and (F) mural cell calcium signaling (dF/F0) in the PA over at least 2 minutes prior to injury and 3-, 7-, 14-, 21-days post-injury in a regression and repair event.

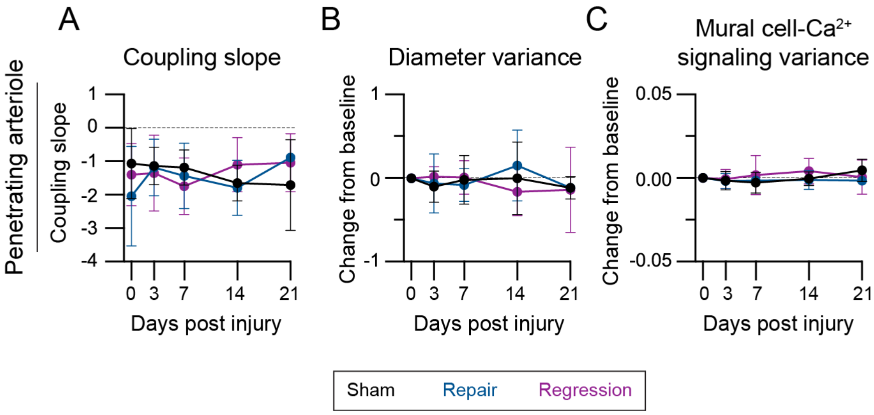


**Supplemental Figure 10. Mural cell-calcium and vessel diameter dynamics are not altered in penetrating arterioles following capillary regression.**
**(A-C)** Graphs of (A) coupling slope, (B) change in diameter variance, and (C) change in mural cell-$Ca^{2+}$ variance over the course of 21 days in penetrating arterioles in sham, repair, and regression events. ANOVA tests detected no significant differences. Sham n=4, repair n=3, regression n=7; 5 mice.

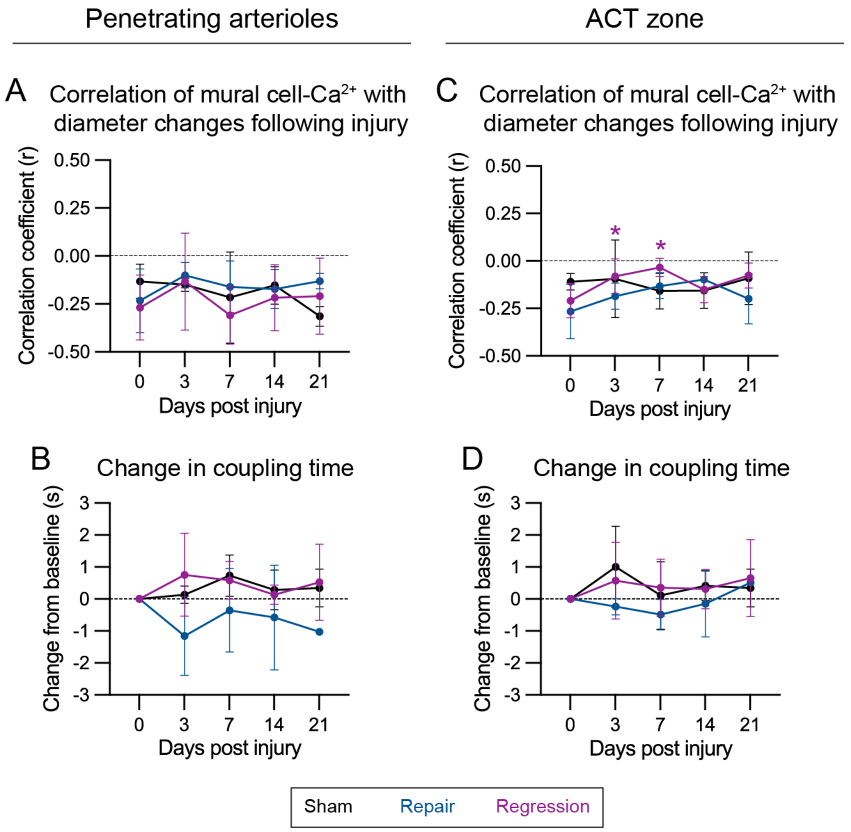


**Supplemental Figure 11. The correlation of mural cell calcium signaling with diameter oscillations decreases after injury in the arteriole-capillary transition zone, while the coupling time remains unchanged.**

**(A, C)** Graphs showing the correlation coefficient (r) of the relationship between mural cell calcium signaling and vessel diameter oscillations in the (A) penetrating arteriole and (C) arteriole-capillary transition (ACT) zone upstream of sham (black), repair (blue), and regression (purple) events over the course of 21 days. ANOVA followed by Dunnett's multiple comparison tests were performed: Correlation of mural cell-$Ca^{2+}$ signaling with diameter - Regression: 0 vs. 7 days: *p=0.027, 0 vs. 14 days: *p=0.03. Sham=4, repair n=3, regression n=7; 5 mice.

**(B, D)** Graphs showing the change from baseline in the coupling time between mural cell calcium signaling and vessel diameter oscillations in the (B) penetrating arterioles and (D) ACT zone upstream of sham (black), repair (blue), and regression (purple) events over the course of 21 days. ANOVA tests detected no significant differences.

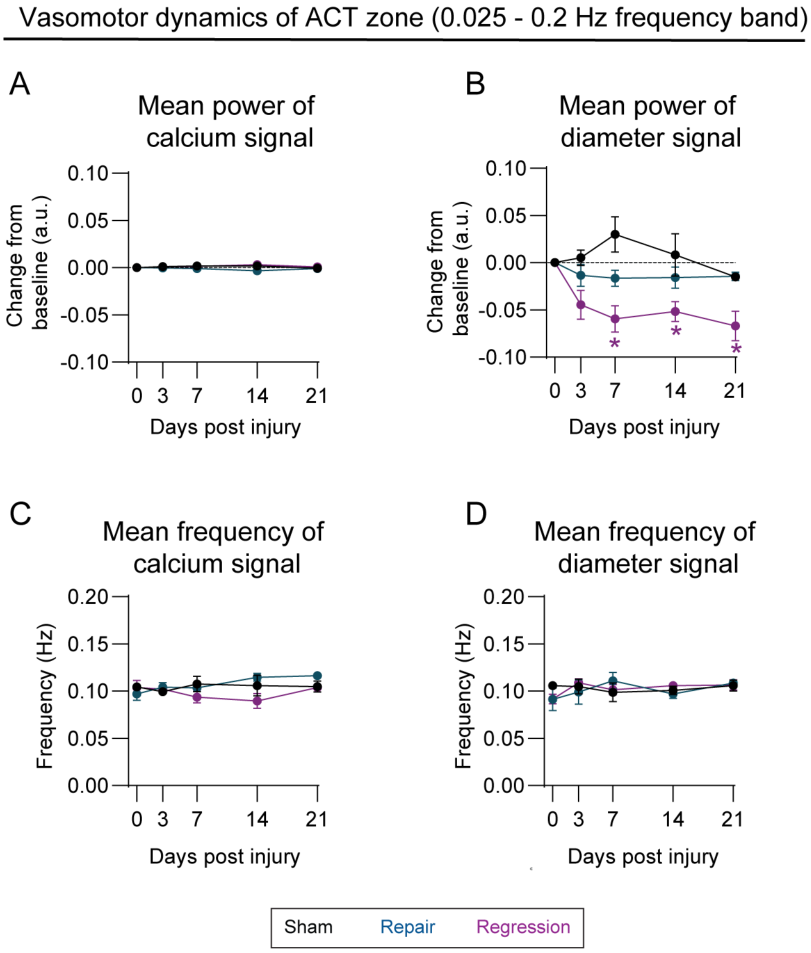


**Supplemental Figure 12. Frequency and power of mural cell calcium signaling in the 0.025 - 0.2 Hz frequency band is maintained in the arteriole-capillary transition zone.**

**(A, B)** Graphs showing the change from baseline of the mean power of (A) mural cell calcium signaling and (**B**) vessel diameter oscillations in the arteriole-capillary transition (ACT) zone upstream of sham (black), repair (blue), and regression (purple) events over the course of 21 days. ANOVA followed by Dunnett's multiple comparison tests were performed: Mean diameter power- Regression event: 0 vs. 7 days: *p=0.0350, 0 vs. 14 days: *p=0.0218, 0 vs 21 days: *p=0.0344. Sham n=3, repair n=3, regression n=5; 5 mice. Data are shown as mean ± SEM.

**(C, D)** Graphs showing mean frequency of (C) mural cell calcium signaling and (**D**) vessel diameter oscillations in the ACT zone upstream of sham (black), repair (blue), and regression (purple) events over the course of 21 days. ANOVA tests detected no significant differences. Data are shown as mean ± SEM.
